# Dynamics of mobile genetic elements of *Listeria monocytogenes* persisting in ready-to-eat seafood processing plants in France

**DOI:** 10.1186/s12864-020-6544-x

**Published:** 2020-02-06

**Authors:** Federica Palma, Thomas Brauge, Nicolas Radomski, Ludovic Mallet, Arnaud Felten, Michel-Yves Mistou, Anne Brisabois, Laurent Guillier, Graziella Midelet-Bourdin

**Affiliations:** 1 ANSES, Laboratory for Food Safety, University Paris-Est, Maisons-Alfort, France; 2 ANSES, Laboratory for Food Safety, Boulogne-sur-Mer, France; 3 INRAE, MaIAGE, University Paris-Saclay, Jouy-en-Josas, France

**Keywords:** *Listeria monocytogenes*, Persistent clones, Food processing plant, Comparative genomics, Mobile genetic elements, Horizontal plasmid transfer, Prophage profiling, Genomic island

## Abstract

**Background:**

*Listeria monocytogenes* Clonal Complexes (CCs) have been epidemiologically associated with foods, especially ready-to-eat (RTE) products for which the most likely source of contamination depends on the occurrence of persisting clones in food-processing environments (FPEs). As the ability of *L. monocytogenes* to adapt to environmental stressors met in the food chain challenges the efforts to its eradication from FPEs, the threat of persistent strains to the food industry and public health authorities continues to rise. In this study, 94 food and FPEs *L. monocytogenes* isolates, representing persistent subtypes contaminating three French seafood facilities over 2–6 years, were whole-genome sequenced to characterize their genetic diversity and determine the biomarkers associated with long-term survival in FPEs.

**Results:**

Food and FPEs isolates belonged to five CCs, comprising long-term intra- and inter-plant persisting clones. Mobile genetic elements (MGEs) such as plasmids, prophages and transposons were highly conserved within CCs, some of which harboured genes for resistance to chemical compounds and biocides used in the processing plants. Some of these genes were found in a 90.8 kbp plasmid, predicted to be” mobilizable”, identical in isolates from CC204 and CC155, and highly similar to an 81.6 kbp plasmid from isolates belonging to CC7. These similarities suggest horizontal transfer between isolates, accompanied by deletion and homologous recombination in isolates from CC7. Prophage profiles characterized persistent clonal strains and several prophage-loci were plant-associated. Notably, a persistent clone from CC101 harboured a novel 31.5 kbp genomic island that we named *Listeria* genomic island 3 (LGI3), composed by plant-associated loci and chromosomally integrating cadmium-resistance determinants *cadA1C*.

**Conclusions:**

Genome-wide analysis indicated that inter- and intra-plant persisting clones harbour conserved MGEs, likely acquired in FPEs and maintained by selective pressures. The presence of closely related plasmids in *L. monocytogenes* CCs supports the hypothesis of horizontal gene transfer conferring enhanced survival to FPE-associated stressors, especially in hard-to-clean harbourage sites. Investigating the MGEs evolutionary and transmission dynamics provides additional resolution to trace-back potentially persistent clones. The biomarkers herein discovered provide new tools for better designing effective strategies for the removal or reduction of resident *L. monocytogenes* in FPEs to prevent contamination of RTE seafood.

## Background

*Listeria monocytogenes* is a foodborne pathogenic bacterium responsible for listeriosis, a non-invasive (i.e. febrile gastroenteritis) or invasive (i.e. meningitis and bacteraemia) disease with a statistically significant increasing trend of confirmed human cases in Europe (EU) during the last ten years [1]. Notwithstanding the much lower incidence of infections caused by *L. monocytogenes* (0.48 cases per 100,000 population in 2017) in comparison to *Campylobacter* and *Salmonella enterica*, the high fatality rate (13.8%) of listeriosis (1633 human cases) in the EU in 2017 remains a serious concern for public health authorities [1]. *L. monocytogenes* is mainly transmitted to humans by ingestion of contaminated food, especially RTE food products such as dairy, meat and fish products [2]. The highest percentage of *L. monocytogenes* non-compliance in RTE foods at processing sites was reported in RTE fishery products (3–10%) over 7 years (2008–2015) monitoring [2]. Contamination of RTE products is often linked to the occurrence of strains able to colonize harbourage sites and persist after cleaning and disinfection (C&D) in FPEs [3]. So far there is no full agreement in the scientific community on the definition of *L. monocytogenes* persistence, however, the repeated isolation over a long period of the same subtype from the same FPE has been proposed [3, 4]. Indeed, specific subtypes may persist and/or be reintroduced at different times within FPEs. The persistence of certain subtypes of *L. monocytogenes* in food processing facilities or on equipment has been reported for up to 10 years and linked to food contamination from farm to fork [5]. The persistence of such strains in the food system has also been observed to play a significant role in listeriosis outbreaks during the last decades [6–8].

The establishment of persistent *L. monocytogenes* subtypes in a specific FPE may occur due to introduction(s) of resistant strain(s), and/or adaptation to the selection pressure met in the FPEs. In addition, the adaptation to particular environmental niches may shelter them from sanitizers used in the food industry. Persistence of such subtypes in RTE production premises is most likely promoted by i) food safety noncompliance (e.g. improper hygiene condition, failure to clean and disinfect food equipment, inadequate food facilities, etc.), ii) possible reintroductions of genotypes showing persistence potentials from external habitats or raw materials, iii) recontamination events due to inadequate sanitisation, and iv) the promoted survival and multiplication under sub-optimal conditions in environmental niches [9, 10]. All these factors (either individually or in combination) along with the dynamics of *L. monocytogenes* populations and the complexity of the transmission pathways of persistent and transient strains in FPEs make the identification of the point of exposure source a critical task in risk management, public health preventions and food industry interventions.

The circulation of different *L. monocytogenes* subtypes was observed in food environments and clinical samples [11]. Strains from *L. monocytogenes* lineage II and serotype 1/2a have been more frequently collected in foods and food processing environments than strains from lineage I [12]. Accordingly, *L. monocytogenes* clonal complexes (CCs), defined as clusters of multilocus sequence typing (MLST) that share at least six alleles, have been epidemiologically associated with human listeriosis and with foods, based on the relative frequency among clinical and food-related sources [12–14]. These observations suggest that some *L. monocytogenes* clonal groups might harbour unique genotypic and phenotypic features facilitating their survival and growth in food and FPEs, as well as their potential transmission to humans. Multiple plasmid-borne homologous genes have been recently associated with lineage II food isolates [15]. Without a full description of the causal mechanisms promoting this particular phenotype, strains belonging to food-associated clonal groups of lineage II, including CC121, CC9, CC8, CC101, CC7 and CC204, have been shown to be persistent in FPEs [16–22].

Biofilm-forming ability, physiological adaptation and tolerance to environmental stresses such as temperature, osmotic and oxidative stresses as well as resistance to heavy metals and disinfectants have been phenotypically and genotypically investigated in specific serotypes and CCs of *L. monocytogenes* to elucidate their persistence mechanisms [19, 23–29] and comprehensively reviewed by Bergholz et al. (2018) [30]. Hence, subtype-specific genetic biomarkers contributing to the persistence phenotype have been described. So far Stress Survival Islet 1 (SSI-1) and SSI-2, including gene clusters involved in low pH and high salt concentrations tolerance as well as in alkaline and oxidative stress response, have been identified in *L. monocytogenes* predominantly belonging to serotypes 1/2c, 3b, 3c and to CC121 [31–33]. A recent study of Hingston et al. (2017) associated the presence/absence and variations of genetic biomarkers such as the virulence gene *inlA* to different levels of cold and desiccation tolerance in specific serotypes of *L. monocytogenes* [25]. Tolerance to disinfectants based on quaternary ammonium compounds (QACs), such as benzalkonium chloride (BC), and heavy metals, such as cadmium (Cd) and arsenic (As), have been described in persistent and presumably transient *L. monocytogenes* strains in FPEs [19, 23, 27, 28, 34, 35], as well as linked to specific molecular mechanisms. For instance, the three-gene cassette *bcrABC*, located either on the chromosome or transposable units of *L. monocytogenes* strains, was described as conferring an increased resistance to BC, a widely used QAC disinfectant in the food industry in the past few decades [36]. The *bcrABC* cassette was detected in *L. monocytogenes* strains belonging to CC121, CC5, CC9, CC8, CC14 and CC204 [19, 21, 26] but not to CC7, CC101 and CC155 [18, 26]. In addition, the transposon *Tn6188* carrying the transporter QacH, and the transposon LGI1, carrying a small multidrug-resistant (SMR) efflux pump encoded by *emrE*, both responsible for enhanced BC tolerance, have been described as specific to CC121 and CC8 strains, respectively [35, 37, 38]. Recommended levels of QACs concentrations in the food industry are much higher than resistance levels conferred by these genes [39] and highly effective against the growth of planktonic bacteria [40]. However, the ability to grow in complex surface-associated communities in the form of biofilms has been shown to play a role in the protection of certain *L. monocytogenes* strains [35, 41] and CCs (e.g. CC121, CC9, CC204) [20] on food processing plant surfaces, enhancing cells persistence potentials in the food industry. Several Cd-resistance determinants were described in *L. monocytogenes* chromosome (e.g. *cadA3*) and plasmids (e.g. *cadA1* and *cadA2*) [42, 43], often together with putative copper resistance determinants and co-selected with QACs resistance efflux pump, as the case of a *cadA2*-harbouring plasmid [44]. The recently described *cadA4* determinant [29] has been identified in the As-resistance island LGI2 of *L. monocytogenes* human strain Scott A and other *L. monocytogenes* serotype 4b as well as in few persistent strains belonging to CC14 and CC204 of Lineage II [17, 19, 45].

As results of these studies, the persistence of certain *L. monocytogenes* subtypes is more commonly considered to arise from a complex combination of several factors rather than a single genetic or individual trait [3, 23, 46]. Hence, understanding the genome-wide diversity and dynamics of MGEs in relevant *L. monocytogenes* CCs for the food industry is crucial to gain useful information to disentangle their persistence in FPEs. Based on whole genome sequencing (WGS), this can be currently achieved by investigating single nucleotide polymorphisms (SNPs) at the core level (i.e. genomic sequences conserved across the whole population) and the presence/absence of genes at the accessory level (i.e. genes only present in subgroups of the population). Furthermore, investigating accessory genes enriched in strains under particular environmental conditions may help to unravel their adaptation mechanisms. The purpose of this study was, therefore, to explore the genomic diversity across *L. monocytogenes* subtypes repeatedly isolated for 2 up to 6 years from food and FPEs samples collected in three seafood processing facilities closely located in the French region of Boulogne-sur-Mer. The main aims were to determine highly-related persistent clonal strains and identifying genetic biomarkers that can be used to predict their adaptation and long-term survival in food-processing facilities, using a combination of de novo whole-genome analyses.

## Results

### Distribution of CCs in persisting *L. monocytogenes* isolates from three French seafood facilities

A comparative genomics analysis was performed in this study based on a selection of 94 *L. monocytogenes* isolates belonging to the prevalent pulsotypes repeatedly collected over time from food and FPEs of the three different French RTE seafood processing plants (A, B, C) (Additional file 1).

**Fig. 1:**
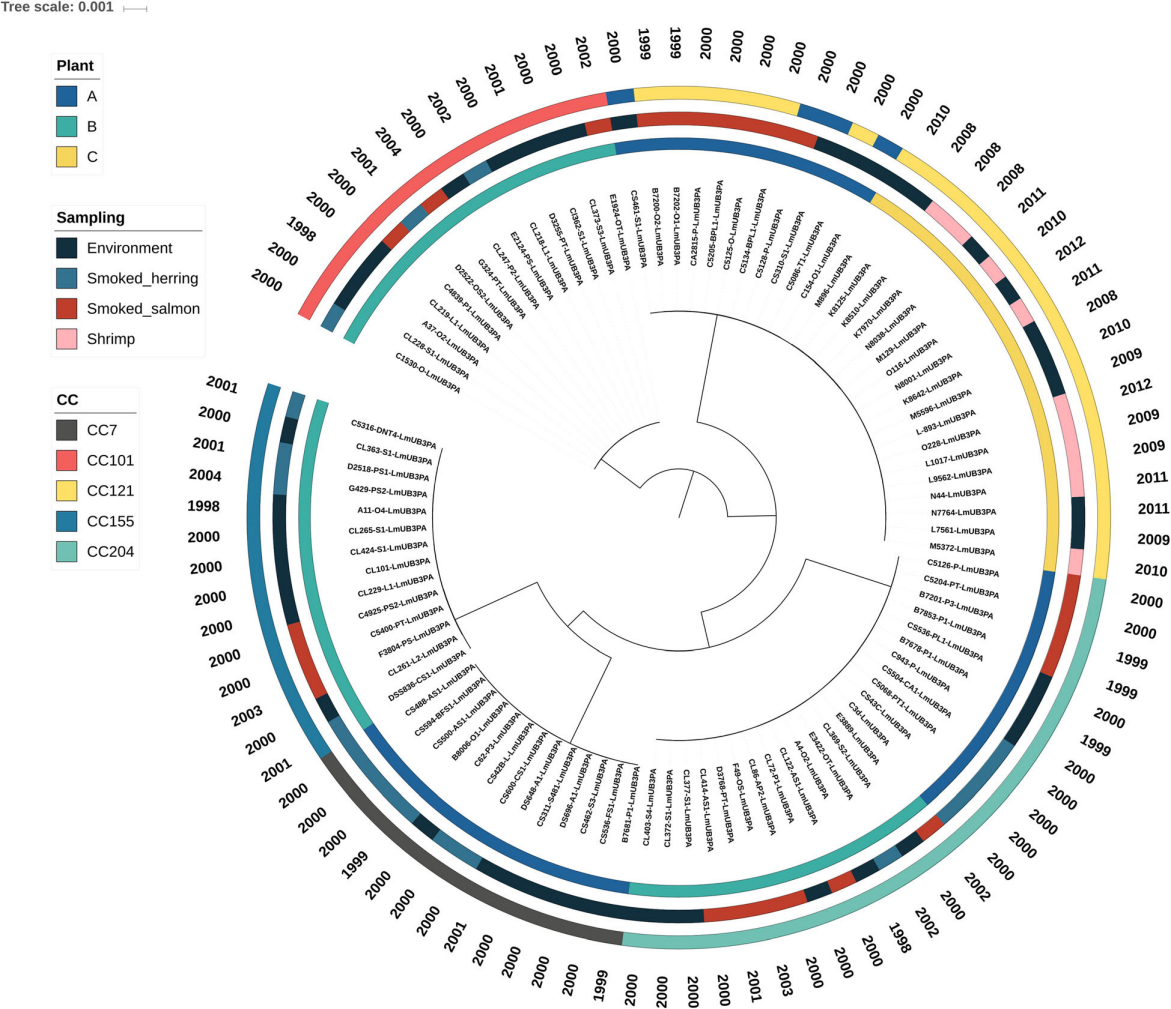
SNPs-based phylogenomic reconstruction. The phylogenetic structure of the five *L. monocytogenes* CCs from the food processing plants was visualized on iTOL (https://itol.embl.de/). The maximum-likelihood phylogenomic reconstruction is based on 50,349 core genome SNPs extracted using iVARCall2 from 94 genomes. The originating processing plant, source, CC and date of isolation are shown on the tree (from inner to outer circles)

In accordance with previous studies [3, 4, 46], the selected *L. monocytogenes* genotypes (i.e. multiple isolates with indistinguishable PFGE profiles) were considered as putatively persistent since isolated over 6 months from the same or different source (e.g. RTE products and FPEs) within the same facility. The 94 food and FPEs isolates of *L. monocytogenes* CCs were recurrently collected over 2 to 6 years from smoked-herring and smoked-salmon producing plant A (*n* = 35) and B (*n* = 41), as well as shrimp processing plant C (*n* = 18). After reads processing through ARTwork [47], two isolates out of the 96 sequenced were excluded because of suspected contaminations (Additional files 2 and 4). An overview of the draft genomes quality, i.e. genome length, N50 values, GC content and number of genes CDSs, including mapping parameters, i.e. depth and breadth of reads coverage, of the 94 *L. monocytogenes* isolates is reported in Additional file 3. In accordance with the typical range previously described for *L. monocytogenes* genomes [17, 48–50], the genome size ranged between 2.92 and 3.36 Mbp with a GC content of 37.8%. The number of coding genes (i.e. coding DNA sequences; CDSs) varied from 2810 in the smallest genomes to 3100 in the largest ones. Genomes size variation appear most likely impacted by the presence/absence of accessory genetic elements like plasmids, prophages and transposons harboured by ~ 70%, ~ 56% and ~ 51% of isolates, respectively. Based on the 7-loci PubMLST schema, different *L. monocytogenes* CCs were found to co-exist into the different seafood processing plants (Fig. 1). Isolates collected over at least 15 months from plant A were classified as CC7 (*n* = 13), CC121 (*n* = 11) and CC204 (*n* = 11), whereas isolates collected over nearly 6 years from plant B were assigned to CC101 (*n* = 14), CC155 (*n* = 14) and CC204 (*n* = 13). In contrast, the 18 isolates from the shrimp producing plant C belonged only to CC121 and were isolated during a 4-year sampling period (2008–2012) different than the time of isolation in plant A (1998–2001) and B (1998–2004).

### Phylogenomic clustering and pairwise SNP differences of phylogroups revealed intra- and inter-plant persisting clones

To untangle the genetic relationships of the 94 *L. monocytogenes* strains belonging to different CCs but isolated in the same environment for a long timeframe (over 2–6 years), an assembly-free core genome SNPs-based strategy was applied [51]. The Maximum-Likelihood (ML) phylogenomic tree was built on the concatenated core genome variants consisting of 50,349 SNPs identified among the 94 genomes (Fig. 1). Overall, the core genome SNPs-based phylogenomic reconstruction showed that the clusters of genomes correspond to the CC-types rather than with the sources or years of isolation (Fig. 1).

**Fig. 2:**
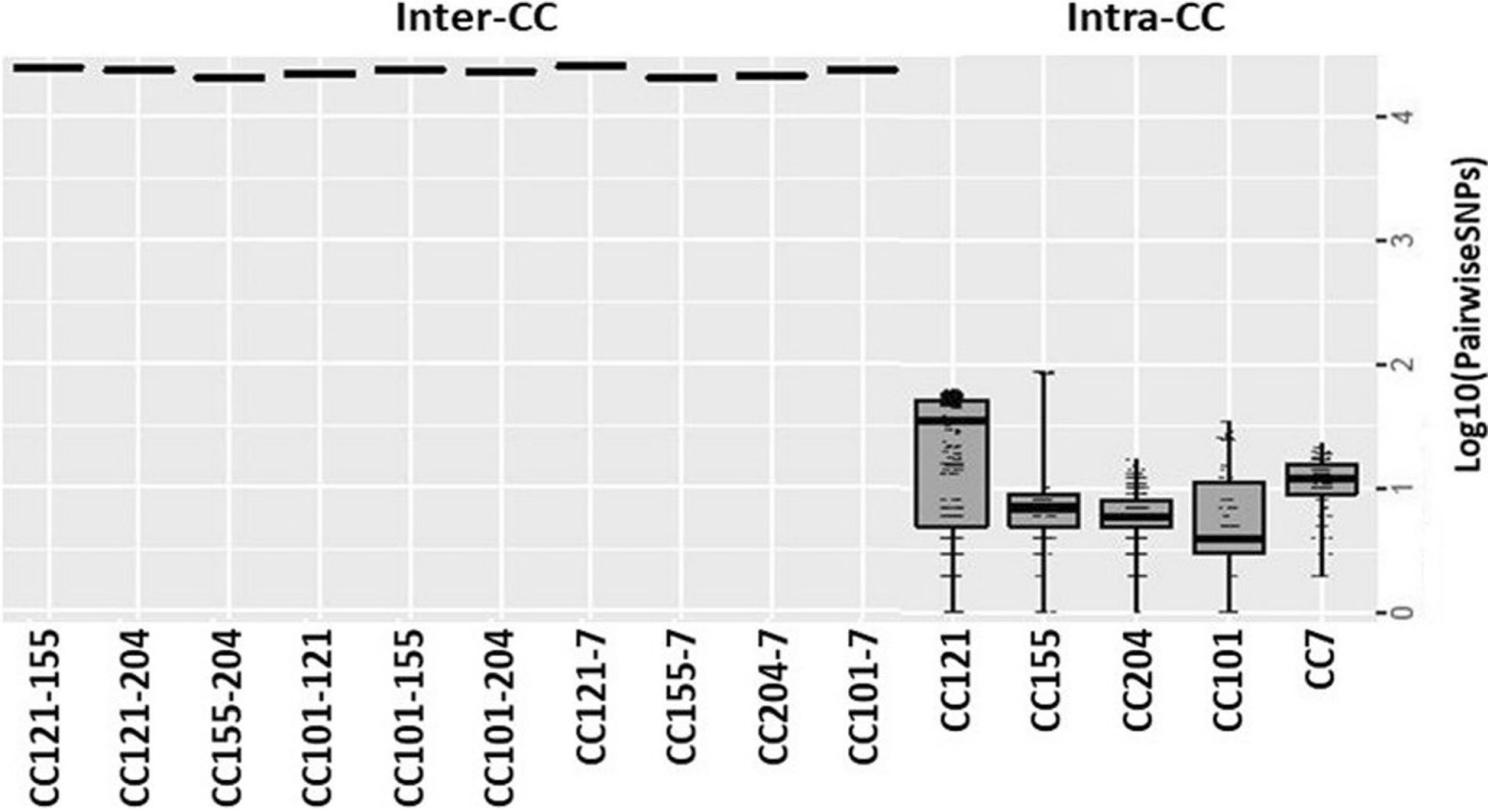
Pairwise SNP distances. Boxplots of pairwise SNP distances computed within and between strains from different *L. monocytogenes* CCs

Albeit the isolation time spanned several months or years, the pairwise SNP distances between the isolates from individual CCs (intra-CC) were limited (highest mean 30 SNPs, range 0–63). In contrast, expected high pairwise SNP differences (ranging from 20,451 to 25,534) were observed between CCs (inter-CC) (Fig. 2). The highest genetic diversity was detected in CC121 (i.e. 1st and 3rd quartiles of log_10_ pairwise SNP distances = 0.69 and 1.7, corresponding to 5 and 51 SNPs, respectively). Nevertheless, considering the CC121 strains from plant A and B separately, much lower levels of pairwise SNP differences were observed. For instance, 10 out of 11 isolates from plant A were characterized by pairwise distances ranging from 2 to 25 SNPs while the pairwise difference of the remaining isolate (CS461-S1-LmUB3PA) ranged from 28 to 61 SNPs. Moreover, the 18 isolates collected over 4 years from plant C were remarkably genetically close with an overall pairwise SNP difference from 0 to 8. A similar pairwise difference (0–10 SNPs) was found between CC155 isolates collected from plant B during 6 years (1998–2004), with the only exception for the DSS836-CS1-LmUB3PA strain showing a median pairwise distance of 86 SNPs. Likewise, the pairwise SNP distances between 12 out of 14 CC101 isolates collected from the same processing plant in a 4-years period (2000–2004) was very low (0–8). Interestingly, no SNP differences were observed between four of these isolates collected from different food matrices (i.e. smoked-salmon and smoked-herring) and the FPEs of plant B. The most genetically distant strains within CC101 were A37-O2-LmUB3PA and C1530-O-LmUB3PA, collected in 1988 and 2000, with a maximum pairwise difference of 15 and 34 SNPs, respectively. The genetic variation of CC204 isolates was likewise limited (0 to 17 SNPs), with a maximum of 10 SNPs between strains collected over 5 years (1998–2003) from plant B. Interestingly, less than 10 SNPs were also detected between CC204 isolates collected years apart in plant A and B from RTE foods and FPEs samples. Slightly higher levels of genetic diversity were found between CC7 isolates in comparison to the other clonal groups with the 1st and 3rd quartiles of 0.9 and 1.19, respectively (Fig. 2). However, these isolates were collected over several months in processing plant A and showed a maximum pairwise distance of 23 SNPs.

### Pangenomic extraction and clustering of accessory genes reveal CC-specific genetic traits

Given the highly conserved core genome, a pangenome analysis was performed on the 94 genomes to investigate the genetic dynamics in terms of locus content within and between the phylogenetically shaped CCs. The pangenome was extracted with Roary [52] from the Prokka-annotated GFF3 files [53] and consisted of a matrix of 4935 group of orthologues of which 2526 core genes (i.e. genes shared by the 99% of isolates). The 2409 accessory genes included 29 soft-core genes (i.e. genes in 95% ≤ strains < 99%), 976 shell genes (i.e. genes in 15% ≤ strains < 95%) and 1404 cloud genes (i.e. genes in 0% ≤ strains < 15%). These observations are consistent with previous comparative genomics studies unravelling the *L. monocytogenes* pangenome [48, 49, 54, 55]. From the Phandango interactive visualization of the accessory genes based distance tree, associated metadata and the pangenome matrix (Additional file 6), five major clusters representing the CCs of *L. monocytogenes* genomes were identified. As observed in the SNP-based phylogenomic reconstruction, strains gathered within each cluster independently of the source or year of isolation. Most of *L. monocytogenes* genomes are conserved between CCs, however, a cluster distribution of CC-specific accessory traits was observed (Additional file 6). A preliminary screening of these accessory traits showed MGEs possibly explaining the successful adaptation of specific sub-clones to FPEs. For instance, putative prophage- and transposon-related clusters of genes were identified in the accessory matrix and appeared to be the major loci of genetic diversity across different CCs. Moreover, the intra-CC variations of the accessory genes content discriminated with higher resolution the genetic diversity observed in the core genome SNPs (Additional file 6). Gene clusters playing an important role in the survival of *L. monocytogenes* cells under the suboptimal conditions encountered in FPEs were detected in individual CC but also shared between different CCs. For instance, a transposon harbouring Cd- and As-resistance cassettes was conserved in all CC204 isolates from this study and inserted in the *yfbR* gene. This genomic island showed high homology (99% average nucleotide identity (ANI)) to *TnyfbR*, a large (~ 35.7 kbp) transposon inserted in the *yfbR* gene and recently identified in CC204 strains isolated from processing environment [17], as well as to the *Listeria* genomic island 2 (LGI2), previously described in the *L. monocytogenes* ScottA outbreak strain [45]. It contains the cadmium-resistance gene *cadA4*, conferring resistance to 35 mg/L of Cd chloride [29], and an As-resistance cassette including multiple loci (i.e., *arsA-1, arsA-2*, *arsR*, *arsD-1, arsD-2*, *acr3*). Additional genetic features such as transcriptional factors and an FtsK domain protein are also included in this genomic island. All CC121 isolates from plant A and B harboured the *Tn6188* transposon, a *Tn554*-like transposon already described in CC121 strains as responsible for enhanced tolerance to BC [38, 56]. This genetic island includes a TetR/AcrR family transcriptional regulator, three consecutive transposase genes (*tnpABC*) and the *qacC* gene encoding for an SMR efflux pump involved in the extrusion of QACs [57]. The stress survival islet (SSI-1) [31] and the plasmid-borne *brcABC* efflux pump [24], respectively associated to acid/salt stress and to BC enhanced tolerance in FPE, were identified in genomes mainly from the FPEs and belonging to CC204, CC7 and in a sub-cluster of CC155. These observations suggest that persisting clones might acquire, conserve and possibly transfer specific MGEs as results of adaptive processes to FPE-associated stressors.

### Contribution of plasmidome and mobile elements to FPE-associated stress survival

The plasmidome of the 94 *L. monocytogenes* genomes were de novo reconstructed and the gene content investigated to untangle the MGEs contribution in the long-term survival of clonal groups. Combining plasmidSPAdes [58] and MOB-suite [59] algorithms, different plasmids were assembled and further compared with a comprehensive plasmid database (Table 1, Additional file 7).

**Table 1:** In silico reconstruction and typing of the plasmidome and comparison with publicly available sequences from public databases NCBI and PLSDB. The plasmids reconstructed by plasmidSPAdes [58] and MOB-recon [59] are reported for each *L. monocytogenes* CC with predicted mobility based on the MOB-typer module [59]. Only the public sequences with ANI > 99.9% and mash distance < 0.002 were reported from NCBI and PLSDB databases [60], respectively

CC	plasmidSPAdes	MOB-recon	MOB-typer	NCBI (ANI > 99.9%)	PLSDB (mash dist < 0.002)
121	62.2^a^ (22; 75%)^b^	61.1 (27; 93%)	conjugative	pLM6179	pLM6179/ pGMI16–004/ pCFSAN022990
7	81.6 (12; 92%)	81.6 (12; 92%)	mobilizable	pLM80	pCFSAN004330/ pLIS1/ pN1-011A/pCFSAN021445
204	90.8 (22; 92%)	90.8 (22; 92%)	mobilizable	pLM80	pN1-011A/ pCFSAN021445/ pCFSAN004330/pLIS1
155	90.8 (6; 43%)	90.8 (6; 43%)	mobilizable	pLM80	pN1-011A/ pCFSAN021445/ pCFSAN004330/pLIS1

^a^*Plasmid size in kbp;* ^*b*^*Number of positive isolate and corresponding percentage in brackets*

**Fig. 3:**
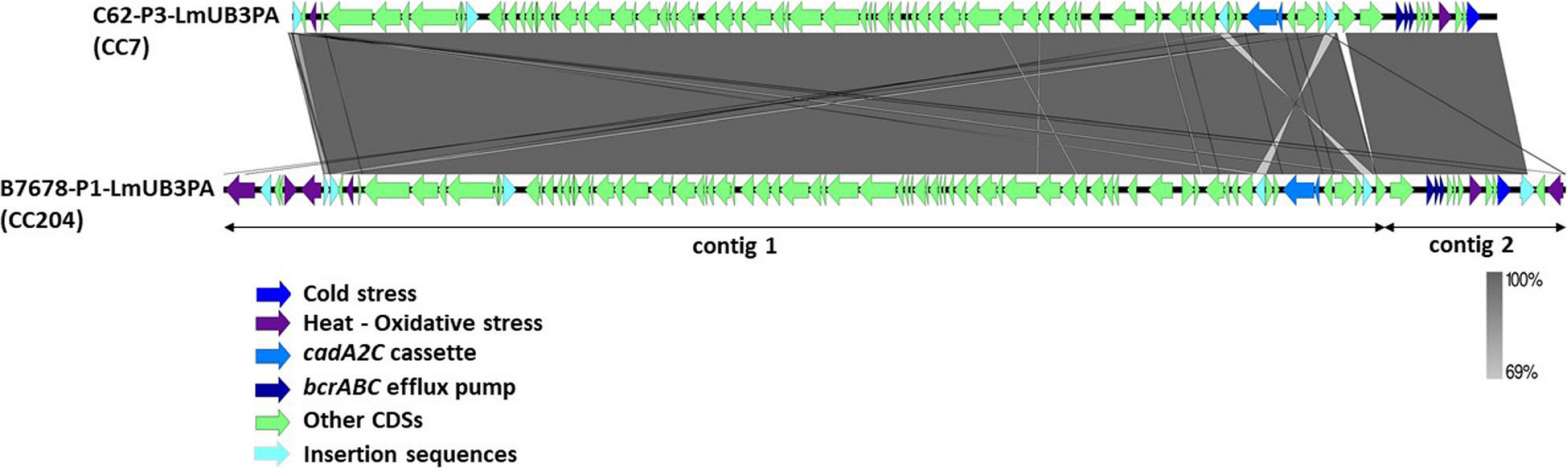
Alignment of plasmid sequences from CC7 and CC204 strains. BLAST-based alignment of the closed 81.6 kbp plasmid reconstructed from CC7 strain C62-P3-LmUB3PA and the 2 contigs 90.8 kbp plasmid reconstructed from CC204 strain B7678-P1-LmUB3PA. Predicted CDSs are represented by arrows with CDSs relevant for *L. monocytogenes* stress response in FPEs coloured as in legend. Nucleotide identity levels (from 63 to 100%) are indicated in grey shaded regions by colour intensity

A large 90.8 kbp plasmid sequence (split into two contigs) was shared by ~ 92% of CC204 food and FPEs isolates (*n* = 22) from plant A and B, and ~ 43% of CC155 (*n* = 6) isolates. Interestingly, all but one plasmid-harbouring isolates from CC155 were from the FPEs of plant B. Although conserved across different CCs, the 90.8 kbp plasmids were found almost identical (99.9% ANI) in CC204 and CC155 strains collected in plant B from 2000 to 2003. On the other hand, the CC204 food and FPEs isolates from plant A were already plasmid-positive in the first sampling in 1999. A smaller sequence of 81.6 kbp was reconstructed in a single contig across ~ 93% of food and FPEs isolates from CC7 (*n* = 12). This plasmid was almost completely aligned with to the 90.8 kbp plasmids showing high similarity (99.9% ANI) over ~ 99% of the sequence. The plasmid from CC7 appears to be the result of a deletion of around 9 kbp sequence (Fig. 3), including oxidative stress-response CDSs (e.g. *qorB* and *RavA* ATPase; see Additional file 8 for more details), and a recombination event with the plasmid from CC204 (Fig. 3). None of the plasmid sequences was found in two CC7 isolates from plant A as well as two CC204 isolates, one from each plant (A and B). All these isolates were collected at the beginning of the sampling timeframe (1998–1999). A total of 98 and 89 CDSs were predicted for the 90.8 kbp and 81.6 kbp plasmids, respectively, including genes implicated in stress response such as Cd-resistance determinants *cadA2C* and the efflux pump *bcrABC* conferring resistance to BC (Fig. 3).

One of the first plasmid harbouring the *cadA2C/bcrABC* resistance markers was pLM80 from the *L. monocytogenes* strain H7858 responsible for the 1998–1999 hot dog outbreak [24]. High degrees of identity (> 99.9% ANI over ~ 96% coverage) were identified between plasmids from CC7, CC204 and CC155 plasmids and the 82.2 kbp pLM80 (2 contigs: NZ_AADR01000010, NZ_AADR01000058). Therefore, we considered them as pLM80-like plasmids. The pLM80-like plasmids showed high identity (99.9% ANI) also with highly conserved plasmids described in ST204 strains from food products and FPEs in Australia and Ireland [17], as well as in ST5 and ST204 strains from an Austrian cheese processing facility [21]. Interestingly, the time of sampling of these published genomes spans from 2000 to 2012.

A highly conserved 62.2 kbp plasmid was also assembled in more than 75% (*n* = 22) of CC121 genomes by plasmidSPAdes. This plasmid contained 61 predicted CDSs, which included Cd-resistance determinants *cadA1C*. The CC121 plasmids were closely related (ANI > 99.9%; > 99% coverage) to a 62.2 kbp plasmid named pLM6179 (acc. No. NZ_HG813250.1), previously described in *L. monocytogenes* strains persistent over 8 years (2000–2008) in an Irish farmhouse-cheese plant [22]. Therefore, we considered plasmids from CC121 as pLM6179-like plasmids. In a recent study comparing plasmid-cured strains and pLM6179-harbouring wild types, the latter showed higher tolerance levels in FPE-associated stressors [28], suggesting that the plasmid-positive strains may acquire survival advantages to temperature, osmotic, oxidative stress and disinfectants in FPEs.

Since similar plasmids were conserved within CCs as also spread across multiple CCs, the plasmids mobility was predicted with MOB-suite [59]. The pLM80-like plasmids were classified as “mobilizable” while the pLM6179-like plasmids as “conjugative”. The MOB-reconstructions of plasmids from the draft genomes (i.e. ARTwork assembly) were also performed, retrieving the same number and type of plasmids in CC7, CC155 and CC204 genomes in comparison to plasmidSPAdes. Moreover, MOB-suite reconstructed plasmids from five additional draft genomes of CC121 (total *n* = 27), in comparison to plasmidSPAdes. Exploring the plasmid sequences synteny in the additional positive genomes we further observed that they were identical to the pLM6179-like plasmids but rather than being in a single contig, they were integrated into the chromosome at the *pyrG* locus. *pyrG* is a psychrophilic gene encoding a CTP synthetase previously flagged as functional marker for site-specific integration of plasmids [61]. Combining results from both tools, only 2 environmental strains out of 29 CC121 strains, isolated in 2000 and 2009 from plant A and C respectively, lacked any observed plasmid sequences.

Typical genetic determinants of *Listeria* plasmid group 2, including genes involved in plasmid replication (e.g., *repA*), maintenance (e.g., *parA* and *soj* gene) and putative transfer functions (e.g., type IV secretion system) [42] constituted the assembled plasmids. Genes conferring enhanced tolerance to processing plant-associated environmental stresses (e.g. cold, heat and osmotic) and virulence potential were also identified. See Additional file 8 for a detailed description of the main genetic components of plasmids.

**Fig. 4:**
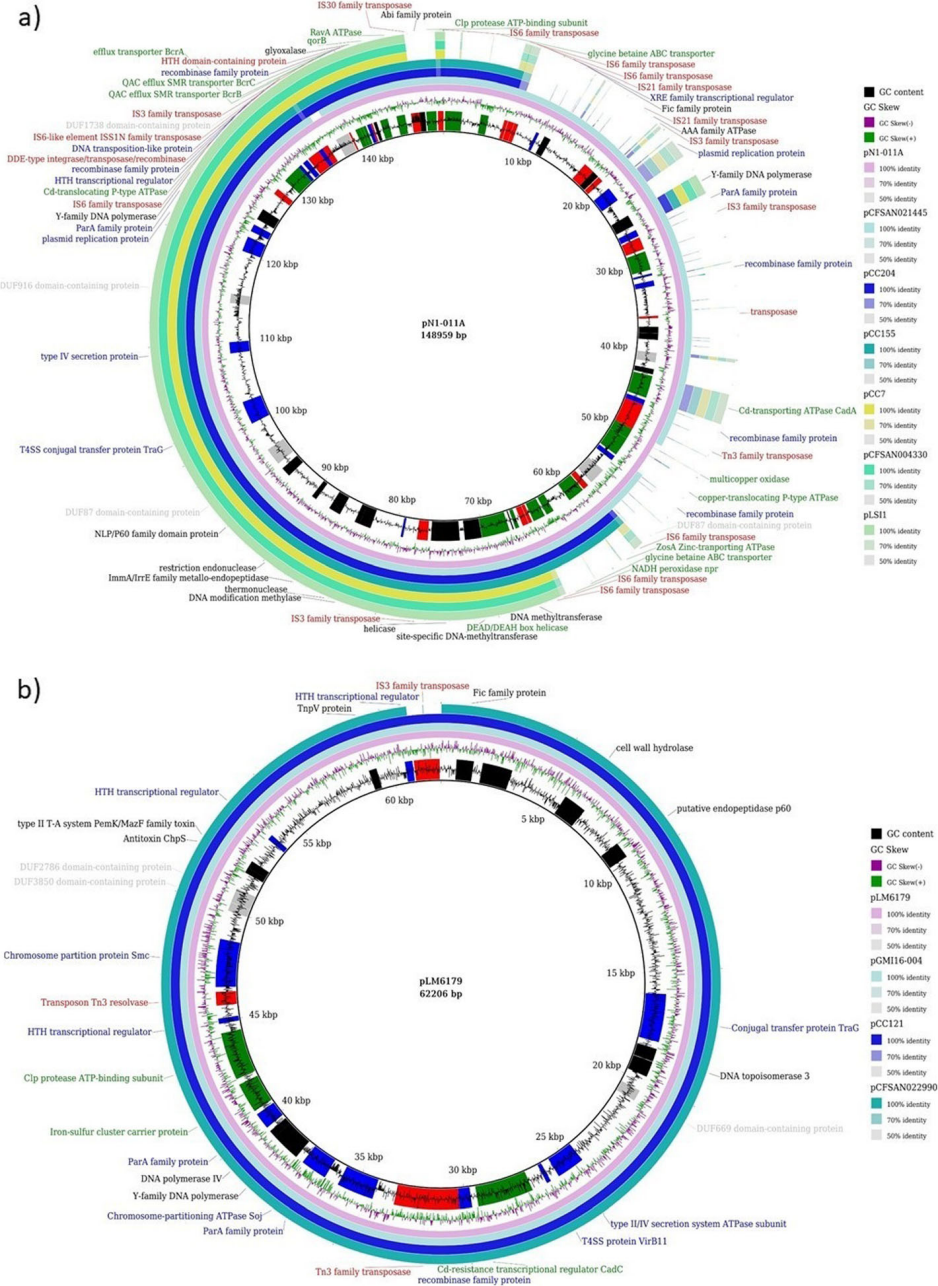
BRIG comparison of the plasmids from this study and the public database PLSDB. Comparison of representative plasmids from CC7 (pCC7), CC155 (pCC155), CC204 (pCC204) (**a**) and CC121 (pCC121) (**b**) isolates with respective genetically closely related publicly available plasmids from PLSDB. The percentage of nucleotide identity refers to the comparison to the reference plasmid pN1-011A (**a**) and pLM6179 (**b**). Externally, CDS annotations are reported excluding hypothetical proteins and coloured based on functions (Additional file 8): transposase (red), stress response determinants (green), plasmid replication, maintenance and transfer (blue), and other functions (black)

The pLM80-like plasmids were genetically close (mash’s distance < 1.77 × 10^− 2^) to different publicly available *Listeria* plasmids (Table 1) from PLSDB plasmid database [60]. In particular, a plasmid from CC7 was nearly identical (ANI > 99%; 100% coverage) to the 81.5 kbp pCFSAN004330 (acc. No. NZ_CP020834.1) and pLSI1 (acc. No. MH382833.1). The CC204 and CC155 plasmids showed higher similarity (ANI > 99%; 100% coverage) to the ~ 150 kbp pN1-011A (acc. No. NC_022045.1) and pCFSAN021445 (acc. No. NZ_CP022021.1) in comparison to pCFSAN004330 and pLSI1 (91% coverage) (Fig. 4 a). Except for pLSI1, all publicly available plasmids belonged to *L. monocytogenes* strains collected from food product and environment in the USA. In contrast, the pLSI1 plasmid belonged to the *L. welshimeri* strain 40/07 isolated in 2007 from sausage in Poland [62]. Korsak et al. (2019) described the *L. welshimeri* strain 40/07 as BC- and Cd-resistant, as well as the *bcrABC*/*cadA2-*harbouring plasmids as potentially highly transferable [62]. These authors also defined the ~ 150 kbp pN1-011A and pCFSAN021445 as composite plasmids resulting from the recombination of a pLSI1-like plasmid and a small *cadA1*-harbouring plasmid similar to pl2015TE24968 from *L. monocytogenes* strain 2015TE24968 (acc. No. CP015985).

Two publicly available *L. monocytogenes* plasmids, the 62.2 kbp pGMI16–004 (acc. No. NZ_CP028184.1) and the 67 kbp pCFSAN022990 (acc. No. NZ_CP020829.1), were reported as genetically close (mash distance < 2.41 × 10^− 3^) to the pLM6179-like plasmid from CC121 with a 99% ANI and 99–97% coverage. Interestingly, pGMI16–004 was found in the *L. monocytogenes* strain CFSAN054109 (GenBank acc. No. GCA_003030165.1) collected in Denmark from smoked fish in 2015.

### Unique prophage profiles characterize persisting clones

The prophage (φ) profiles of the 94 *L. monocytogenes* genomes were characterized using the PHASTER tool [63] for identification and annotation of putative prophage sequences and the integration loci. Prophages or phage-derived elements were detected in all the *L. monocytogenes* genomes included in this study (Additional file 7). To deeply assess genetic diversity within clonal strains, presence and absence of questionable and intact regions, as well as conservation, ANI and integration site of the prophage sequences were considered. A total of 133 intact and questionable prophage regions were identified by PHASTER across 76 genomes from plant A and B. The 18 CC121 isolates from the shrimp-processing plant C harboured only incomplete prophage regions. The region size, phage components, insertion site, number of CDSs, name and percentage of the major phage organism, as well as the GC content were listed in Additional file 9. Besides the chimeric nature of these regions, a major *Listeria* phage organism (homologous to more than 50% of the total number of CDS of the region) was identified in the majority (> 78%) of the prophage dataset. The most commonly identified *Listeria* phages were: A600 [NC_009815], A118 [NC_003216], vB LmoS 188 [NC_028871] and LP-101 [NC_024387]. However, homologies to phages from other bacterial species such as phage 315.2 [NC_004585] from *Streptococcus pyogenes* were also reported (Additional file 9). PHASTER identified unique sets of prophages characterising strains from each clonal group, which presented from one (e.g. CC204, CC155) to three prophages per genome (e.g. CC7 and CC101) mostly integrated within tRNAs or *comK* genes (Table 2, Additional file 9). Within clonal groups, strains harboured highly conserved sequences in terms of number of identified regions and homologous CDSs, genes order and integration loci; although variants of the corresponding prophage length were observed (Table 2, Additional file 9). However, the alignment of these prophages showed high sequence similarity to each other (100% ANI) (Additional file 10). The majority of prophages detected in the *L. monocytogenes* genomes were integrated into the chromosome and four insertion sites were identified: tRNA-Arg (ccg), tRNA-Arg (tct), tRNA-Ser (cga) and *comK* (Table 2).

**Table 2:** Prophages and phage profiles of *L. monocytogenes* CCs gathered by integration hotspots

	CC121	CC7	CC204	CC101	CC155
tRNA-Arg(ccg)	49.9^a^ (10)^b^				
tRNA-Arg(tct)				51.6 (14)	
tRNA-Ser(cga)		33.2 (10); 73.0 (3)		36.9 (14)	
*comk*		50.7 (13)	44.4 (24)	44.5 (14)	46.8 (13)
-^c^		22.1 (13)			

^*a*^*Prophage size in kbp;* ^*b*^*Number of positive isolates,* ^*c*^*Extrachromosomal phage.*

The only exception was a small 22.1 kbp phage homologous to vB LmoS 188, which was conserved as a single contig in all draft genomes from CC7. Interestingly, a high similarity (i.e. more than 92% ANI over 82% of sequence) was observed between this region and φ*comK* (i.e. a prophage mainly homologous to vB LmoS 188) in genomes from CC101 and CC155 (Additional file 9). Moreover, the alignment of φ*comK* found in CC101, CC155 and CC204 showed high ANI values (above 94%) over ~ 70% of the sequences. Isolates from these CCs simultaneously occurred in plant B. Only one-third of the φ*comK* sequence conserved in genomes from CC7 (homologous to phage A118) was aligned to the φ*comK* sequence with an ANI higher than 95% (Additional file 9). However, this sequence showed high nucleotide identity (~ 90%) for almost half of the φtRNA-Arg (ccg) sequence (also homologous to phage A118) detected in genomes from CC121 isolated in the same plant (Additional file 9 and Additional file 10, Table 2). No similarity was detected between φtRNA-Arg (ccg) and both tRNA-integrated prophages detected in all CC101 genomes. However, φtRNA-Ser (cga) from CC101 showed high similarity (i.e. ANI > 96.6%; 84% coverage) to a φtRNA-Ser (cga) conserved in all but 3 genomes from CC7. These φtRNA-Ser (cga) presented a highly mosaic structure with considerably lower similarity to *Listeria* phages in comparison to the other prophages and included CDSs homologous to *Streptococcus* phage 315.2 (Additional file 9). The remaining 3 genomes from CC7 harboured a longer version (73.0 kbp vs 33.2 kbp) of φtRNA-Ser (cga) including around 50 additional phage-related CDSs mainly homologous to *Listeria* LP-101 phage (Additional file 9). Interestingly, CS461-S1-LmUB3PA and DSS836-CS1-LmUB3PA genomes presented different prophage profiles in comparison to other isolates from persisting clones in plant A (i.e. CC121) and B (i.e. CC155), respectively, in addition to the higher pairwise distance (i.e. 28–38 SNPs and 83–86 SNPs, respectively). On the other hand, the C1530-O-LmUB3PA genome presented the same prophage profile as the persisting clones from CC101 in plant B, although the relatively high genetic distance (i.e. 25–34 SNPs).

### Pangenome-wide enrichment analysis identified several genetic elements associated with the plant

Accessory gene clusters were statistically associated by Scoary [64] to identify genetic features enriched in clones persisting for long periods in a specific processing plant. Specificity, sensitivity and different *p*-values (e.g. Naïve, Bonferroni and Benjiamini-Hochberg (BH)) were calculated for each accessory gene and used to point out loci strongly associated with each plant (Additional file 11). With a BH value under 2 × 10^− 5^, 513 loci were almost exclusively present (~ 100% specificity) in genomes from plant A (*n* = 204), B (*n* = 250) and C (*n* = 59) (Additional file 11). Among these loci, a huge portion of hypothetical proteins (*n* = 298; 58%) was reported along with transposon- and prophage-related proteins originating from different bacterial species (*L. monocytogenes, Bacillus, Yersinia*) (Additional file 11). Most of these loci (97%) were inherited together and located in different genomic segments while the remaining were spread across the genome. Interestingly several contiguous loci were included in different prophage regions identified in genomes from CC121 and CC7 isolated in plant A (*n* = 145), CC101 and CC155 from plant B (*n* = 91) as well as CC121 from plant C (*n* = 58) (Additional file 11). Even though the majority of plant-associated loci were CCs specific, 26, 18 and 15 phage-related loci were shared by strains from different CCs simultaneously occurring in plant A, B and C, respectively. This is consistent with the similarities observed in the pairwise comparison of prophages described above.

**Fig. 5:**
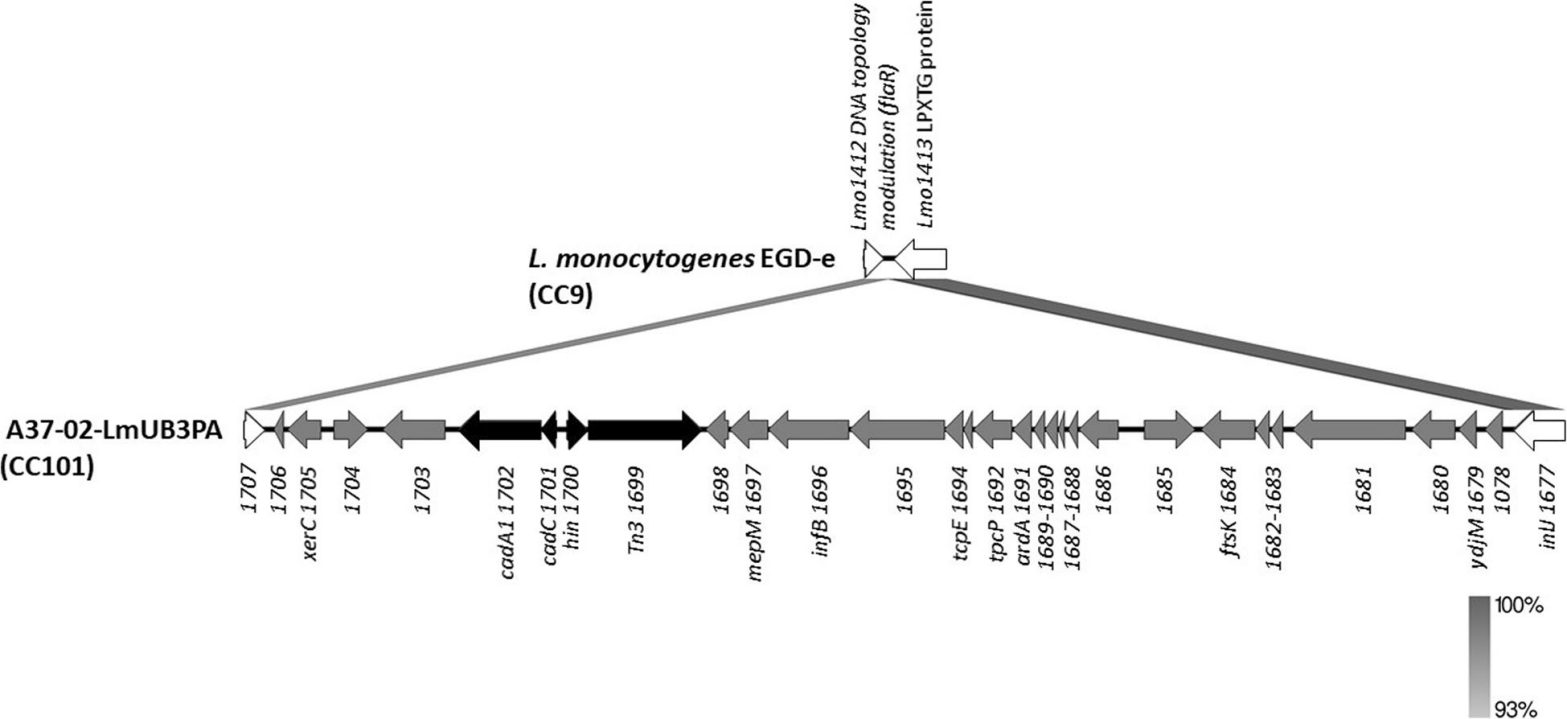
Genomic organization of LGI3 of A37–02-LmUB3PA strain. Within LGI3, genes enriched in plant B and the integrated Cd-resistance cassette are shown by grey and black arrows, respectively, while LGI3-flanking genes in EGD-e strain are shown by white arrows. The Prokka locus tag is reported for each gene

Outside of the prophage regions, a cluster of 25 loci enriched in plant B was found to be part of a large (ca. 31.5 kbp) genomic island that we named *Listeria* genomic island 3 (LGI3). The LGI3 includes 29 predicted CDSs and chromosomally integrates the Cd-resistance determinants *cadA1C*, flanked by the recombinase *hin* and the transposase *Tn3* (Table 3) typical of pLM6179 plasmid. All the genomes containing the *cadA1C*-harbouring genomic island were members of CC101. In these strains, the genomic island is inserted upstream of the internalin gene *inlJ* (*Lmo1413* homolog of strain EGD-e) and includes also genes putatively involved in DNA integration, conjugation, translocation and recombination (Fig. 5, Table 3). In comparison to the whole NCBI database, only the *L. monocytogenes* reference strain ATCC 51775 (acc. No. CP025222.1), isolated from dairy products in Belgium in 1990–1992, showed sequence similarity (99.9% of nucleotide identity) to LGI3. However, the ATCC 51775 sequence lacks the CadAC efflux cassette, suggesting that the 31.5 kbp genetic island is specific to the CC101 clone from this study (Table 3).

**Table 3:** Gene content of LGI3 from nucleotide position 1,653,803 to 1,685,260 of CC101 strain A37–02-LmUB3PA. Loci are reported with Prokka annotation and homologous “*locus tag*” from *L. monocytogenes* reference strains ATCC 51775 for the genes enriched in food the plant (yes) and from *L. monocytogenes* 6179 for the non-enriched ones (no)

Roary gene name	Prokka annotation	Reference strains *locus tag*	Enriched in food plant
group_1188	Hypothetical protein	CXL08_07550	yes
ydjM	Inner membrane protein YdjM	CXL08_07545	yes
group_1190	Hypothetical protein	CXL08_07540	yes
group_1191	Cna protein B-type domain protein	CXL08_07535	yes
group_1192	DUF961 domain-containing protein	CXL08_07530	yes
group_1193	DUF961 domain-containing protein	CXL08_07525	yes
ftsK	DNA translocase FtsK	CXL08_07520	yes
group_1195	Gram-positive cell wall anchor protein	CXL08_07515	yes
group_1196	Replication initiation factor	CXL08_07510	yes
group_1197	Hypothetical protein	CXL08_07505	yes
group_1198	Hypothetical protein	CXL08_07500	yes
group_1199	Hypothetical protein	CXL08_07500	yes
group_1200	Hypothetical protein	CXL08_07495	yes
ardA	Antirestriction protein ArdA	CXL08_07495	yes
group_1202	Conjugative transposon protein TcpC	CXL08_07480	yes
group_723	Hypothetical protein	CXL08_07475	yes
group_1203	TcpE family protein	CXL08_07475	yes
group_1204	Putative conjugal transfer protein	CXL08_07470	yes
infB_1	Translation initiation factor IF-2	CXL08_07465	yes
mepM	Murein DD-endopeptidase MepM	CXL08_07460	yes
group_1207	Hypothetical protein	CXL08_07455	yes
group_1208	Tn3 transposase DDE domain protein	LM6179_p0036	no
hin	DNA-invertase hin	LM6179_p0035	no
cadC	Cd-resistance transcriptional regulatory protein CadC	LM6179_p0034	no
cadA1	Putative Cd-transporting ATPase	LM6179_p0033	no
group_1212	Hypothetical protein	CXL08_07450	yes
group_1213	Helix-turn-helix domain DNA-binding protein	CXL08_07445	yes
xerC_1	Site-specific tyrosine recombinase XerC	CXL08_07440	yes
group_1215	Helix-turn-helix domain DNA-binding protein	CXL08_07435	yes

An additional cluster of 26 contiguous loci involved in carbohydrate transport and metabolism was also statistically enriched in the CC101 persisting clone from plant B. This gene cluster included a number of CDSs specifically involved in the uptake, transport and utilization of primary carbon source (e.g. glucose, fructose, mannose and cellobiose) such as transketolase (tkt) and phosphotransferase system (PTS) operons (e.g. *licABC*). The correlation between the presence of these genes and the response of *L. monocytogenes* to environmental stress in FPEs still needs to be understood, however studies showing the up-regulation of PTS components in *L. monocytogenes* cells under exposure to QACs [65] as well as decreased transcript levels of PTS and related metabolic enzymes under salt stress [66] have been performed. An interesting 9-loci set enriched in plant A included a type I Restriction-Modification (RM) system that involves site-specific recombination within the gene *hsdS* for the specificity subunit (S) and also on/off switches of gene expression [67, 68]. The type I SRM complex is located between genes *LMON_0301* and *LMON_0303* in *L. monocytogenes* strain EGD (acc. No. HG421741), downstream to a recombinase locus (i.e. gene *LMON_0299*). This system has the potential to attack foreign DNA and other competitor RM systems as well as to kill the hosting cell through DNA breakage [68, 69]. All strains belonging to CC7 harboured a type I SRM complex. A ~ 10 kbp genomic region, including a repeat region and clustered regularly interspaced short palindromic repeat (CRISPR)-Cas loci, was also identified as enriched in CC7 genomes and located downstream of a ~ 24 kbp incomplete phage sequence. The CRISPR-Cas loci was constituted by four *cas* genes (*cas9*, *cas1*, *cas2* and *csn2*) upstream of a 1820 bp CRISPR array describing a *Listeria* type II CRISPR–Cas system. CRISPR–Cas loci encode for adaptive defence systems for protecting the bacterial cell from bacteriophage infection and further invading mobile DNA elements [70, 71].

## Discussions

A combination of recently developed bioinformatics tools was applied in this study to assess the persistence phenotype through *L. monocytogenes* CCs contaminating food processing environments. The repeated introduction of the same subtypes in FPEs and/or the ability of certain subtypes to adapt and survive to multiple environmental stressors encountered in FPEs [3, 4] challenge the effort to unravel the genetic basis of *L. monocytogenes* persistence in food processing for decades. The persistence of strains in RTE food producing plants is also of greatest concern when clinically relevant genotypes contaminate the related FPEs. For instances, CC101 and CC155, highly associated with human clinical listeriosis cases [12], represent clonal groups with enhanced pathogenic potential in comparison to CC121 and CC204, mainly found in foods and FPEs [12]. The reasons why some clonal groups of *L. monocytogenes* are recurrently isolated in the FPEs is still under discussion, as well as the dynamics of genetic elements potentially enhancing their persistence. In the last decades, several studies aiming to determine the basis of persistence in RTE industry and to trace-back outbreaks-related *L. monocytogenes* strains have been performed based on conventional molecular typing [72–75]. However, the advantages provided by analyses of genome-scale data through comparative genomic approaches have been recently demonstrated in the context of *L. monocytogenes* strains persisting in FPEs [4, 16, 18, 19, 23, 76–78]. These studies also suggested differences in distribution and fitness of CCs in geographical regions and evolutionary niches. Identify and characterize the CCs recurring in specific processing plants, along with the role of genetic elements contributing to their adaptation to the FPEs stressors may therefore boost the assessment of the *L. monocytogenes* persistence phenotype [30]*.* We tested this hypothesis focusing on the core and accessory genomes of *L. monocytogenes* subtypes repeatedly collected over 2–6 years from food and food-related environments of three closely located plants producing RTE seafood in France.

Persistent strains and newly introduced strains in the FPEs by raw material have been described in relation to specific STs or CCs [18, 20, 78, 79]. With a similar objective, some authors [18, 78, 80] proposed arbitrary thresholds ranging from 4 to less than 10 or 20 until 25 SNPs to identify persisting and “truly persisting” clones of *L. monocytogenes*. Other authors suggested that the isolation of the same *L. monocytogenes* clonal type (CT) at the same sampling point after C&D procedures for a much shorter sampling interval (i.e. less than one month) in comparison to this study was a sufficient parameter to consider *L. monocytogenes* strains as persistent [23]. Our results show that CCs recurring in plants A, B and C represented persisting and even “truly persisting” plant-specific clones including long-term persistent strains with less than 25 pairwise SNPs differences. As already observed in previous studies [18, 78, 79], the repeated detection of the genetically close or even indistinguishable strains from different foods and FPEs during the whole sampling period (2–6 years) bolstered the intra-plant clonality of *L. monocytogenes* strains included in the present study. The only exceptions were single strains for CC155 (DSS836-CS1-LmUB3PA), CC101 (C1530-O-LmUB3PA) and CC121 (CS461-S1-LmUB3PA), considered as transient and likely reintroduced in the FPEs due to the high genetic differences from the persisting clones. Clonal groups of strains from CC121 and CC204 were detected across different plants. Nevertheless, CC121 isolates collected in plant A were genetically distant from the highly clonal isolates colonizing plant C and collected 8 years apart (pairwise differences range: 44–63 SNPs). On the other hand, pairwise difference ranging between 4 and 13 SNPs were found comparing CC204 isolates from plant A and B, suggesting that these strains are from a unique clone that might have been transferred between these two facilities. Some authors concluded that identical or nearly identical (median 2 to 11 SNPs) *L. monocytogenes* strains can occur in different retail deli environments without any links of known transmission [76]. Although the present study confirms this observation, we could also speculate that geographically close companies producing similar foodstuff (e.g. plant A and B) may receive raw seafood from the same suppliers, which would constitute a common source of introduction for potentially persistent *L. monocytogenes* strains. In addition, the presence of the CC204 clone in plants A and B during overlapping sampling timeframe (1999–2001) would support the hypothesis of an inter-plant transmission *L. monocytogenes* strains. This finding is consistent with recent studies highlighting the presence of *L. monocytogenes* clones from ST8, ST9, ST101 and ST14 shared by different meat-processing establishments [16, 78].

The occurrence of persistent intra- and inter-plant clones was corroborated by investigating the accessory genome. A specific repertoire of MGEs (i.e. plasmids, prophages and transposons) characterized strains from individual CC with a high degree of conservation. Previous authors also did similar observation comparing few subtypes of persistent *L. monocytogenes* strains from processing plants [16, 18, 21, 22]. Our study confirmed this finding in a wider diversity of *L. monocytogenes* population showing that strains from additional CCs can acquire and conserve MGEs possibly enhancing their adaptation and survival in controlled FPEs.

Plasmids encoding genes conferring increased tolerance under multiple stress conditions related to food and FPEs were identified in the analysed *L. monocytogenes* from CC121, CC7, CC155 and CC204. These plasmids showed high similarity with globally spread publicly available plasmids harboured by persistent *L. monocytogenes* strains from ST121, ST5 and ST204 isolated in food products and related FPEs [17, 21, 22, 79]. Experimental evidence that such plasmids from *L. monocytogenes* ST121 and ST5 contribute to cells survival under stress condition met in FPEs have been recently proved [28]. Previous authors also suggested the importance of plasmids in the contribution to the survival of *L. monocytogenes* in food and FPEs [16, 17, 21, 22]. However, Naditz et al. (2019) were the first to show it at phenotypic and genotypic levels examining wild type and plasmid-cured strains [28]. In particular, these authors showed that pLM6179 and p4KSM plasmids from ST121 and ST5, respectively, play a key role in the enhanced capacity of *L. monocytogenes* to face some food processing environment-associated stresses like heat, BC, oxidation and osmotic stress. Moreover, Muhterem-Uyar et al. (2018) observed that possible horizontal gene transfer (HGT) of the p4KSM plasmid (also referred as pLM80-like plasmid) took place from strains belonging to dominant ST5 to rare ST204, which later occurred in the same FPE and become the most dominant ST along with ST5. In our study, pLM80 prototypes highly similar to p4KSM were identified in all but one persisting strains from CC204 in plants A and B. Interestingly, this plasmid was identical to a plasmid identified in 6 out of 14 strains from CC155 mainly isolated in FPEs of the plant B. Overall plasmid positive strains from CC204 and CC155 were found over three years in plant B either in FPEs or food samples. Due to the earlier occurrence and higher conservation of the pLM80-like plasmid from CC204 in comparison to CC155 strains, the plasmids have likely been transferred from CC204 to CC155 presumably involving strains colonising the same environmental niche in plant B. A different scenario can be hypothesised in plant A, where a closed smaller plasmid highly similar to plasmids from CC204 was conserved in all but two CC7 strains. The two strains lacking this plasmid were isolated at the beginning of the sampling. Comparison of plasmids from CC204 and CC7 might suggest that plasmids from CC7 might have arisen from recombination of the 2 contigs identified in plasmids from CC204, accompanied by a deletion of around 9 kbp (9 CDSs) pre- or post-transfer from CC204 to CC7. The deleted sequence included several neighbouring genes involved in stress response (e.g., *npr*, *zosA*, *cplB*, *gbuC*) along with three CDSs annotated as insertion sequence (Fig. 3, see Additional file 8). The presence of several insertion sequences points out that plasmids from CC204 are composite plasmids which integrated the genes conferring additional tolerance to plant-associated stresses. However, the high homology observed between plasmid variants from CC204 and CC7 may also suggest that a common ancestral plasmid underwent sequence shuffling and integration/deletion of additional environmental stress response genetic determinants, as also observed by Fox et al. (2016) comparing plasmids from CC204.

Adaptation to specific environmental niches in FPEs and short-term evolution of both distantly and closely related *L. monocytogenes* strains have been linked to the diversification of prophages [50, 79, 81, 82]. ST-specific prophages showing a high degree of conservation in FPEs have been described for strains from ST121 and ST8, while prophages from strains belonging to ST204 presented a lower degree of conservation [16, 17, 22]. Moreover, certain *L. monocytogenes* strains seem to be genetically more prone to be attacked by bacteriophages and transfer prophage genetic material [16]. With CC7 and CC101 showing the highest number of prophage regions per genome, all information herein shows that genetically related strains, isolated at different spots of FPEs over a long time frame, harbour highly conserved prophages in terms of number of identified region, insertion sites and CDSs composition. Interestingly, the same prophage profile characterizes all strains from CC204 in plant A and B while a different prophage profile was observed between strains from CC121 in plant A and C. This supports the hypothesis that phylogenetically shaped CC204 represent an inter-plant clone in contrast to CC121 which includes two intra-plant clones. In addition to the high pairwise SNPs distance, CS461-S1-LmUB3PA and DSS836-CS1-LmUB3PA genomes presented different prophage profiles in comparison to the persisting strains from CC121 and CC155, respectively. These elements support the hypothesis that transient strains (i.e. not persistent) are probably reintroduced by raw material in the processing plants. On the other hand, C1530-O-LmUB3PA genome presented the same prophage repertoire of the CC101 strains, although its pairwise differences from the persisting clone exceeded 25 SNPs. The fact of harbouring the same prophages of persistent strains, including the φ*comK* considered as a driving force of fitness advantages of *L. monocytogenes* in food production environments [82], suggests to also consider this strain as potentially persistent. This observation supports the hypothesis from Fagerlund et al. (2016) and Knudsen et al. (2017) stating that all isolates within an ST or CC present the potential to be or become persistent, especially when sharing the same environment [15, 18].

Previous studies evidenced that φ*comK* are genetic markers involved in the niche-adaptation and persistence of *L. monocytogenes* in meat FPEs, in addition to characterize the genotype diversity within ST (e.g. ST121). According to these studies, the φ*comK* genotypes can be associated to individual food processing operations and allow accurate tracking of persistent strains as well as the design of intervention strategies to reduce the plant contamination [10, 56]. Highly conserved φ*comK* were detected in all the persisting clones analysed in the present study. Small genetic elements, including CDSs associated with the originating plant, were shared between the φ*comK* from different CCs collected in the same FPEs, but also between tRNA-integrated prophages found in different FPEs (Additional file 10). This suggests that possible homologous and/or non-homologous recombination events occurred between different *L. monocytogenes* CCs as also described in previous studies focusing on the persistence of *L. monocytogenes* [16, 76, 82]. However, prophage sequences integrated at the same locus in different strains strongly diverged between plant-specific persisting clones. Moreover, most of the genes significantly enriched in the specific plants were located in distinct prophages, suggesting that these regions include unique genetic markers possibly contributing to niche-specific adaptation of persisting clones.

A cluster of 25 loci significantly enriched in the smoked-fish plant B was found to be included in a novel ~ 31.5 kbp genomic island chromosomally integrating Cd-resistance determinants (*cadA1C*). This region, that we named *Listeria* genomic island 3 (LGI3), is highly conserved and specific to CC101 strains which persisted in plant B over 6 years. Genetic islands in *L. monocytogenes* have been described as MGEs constituted by active integrase for HGT and fitness conferring genes [50]. Although in this study we have no evidence on the HGT of LGI3, the presence of genes putatively involved in DNA integration, recombination and conjugation in this genetic island suggests the possibility of LGI3 transfer to other persisting clones.

## Conclusions

The combination of different bioinformatics solutions evidenced intra- and inter-plant *L. monocytogenes* clones persisting over years in seafood products and processing environments of three French RTE-producing plants. In addition, it provided insights into the dynamics of stress tolerance-related genetic markers promoting the persistence of *L. monocytogenes* CCs in FPEs.

Genetic elements such as prophage loci and LGI3 constitute a genetic signal that may contribute to enhanced adaptation of *L. monocytogenes* persistent clones in FPEs as well as to the identification of their originating site. Functional annotation studies should be performed to fully evaluate the role of these genetic elements in the long-term survival of *L. monocytogenes* clones in food processing plants. However, focusing on genetic markers during surveillance studies can improve the tracking-back of *L. monocytogenes* strains potentially involved in clinical cases. Moreover, the investigation of prophage profiles along with pairwise SNP distances of phylogenetically shaped CCs may provide increased discriminatory power in the identification of persisting clones and inter-plant transmissions.

Genomics studies for tracking the main routes of transmission of *L. monocytogenes* will contribute to define optimal risk mitigation strategies in RTE seafood producing processing plant and support food safety management system in fishery production. Results from these studies would also support stakeholders and risk manager especially as consumption of RTE seafood products increased in recent years. Our results extend evidences from other authors [17, 21, 25] emphasizing that almost identical plasmids conferring enhanced survival against FPE-associated stresses are globally spread on a wider population of phylogenetically distant clones, most likely due to the high selective pressure and availability of these genetic elements in FPEs. Constant environmental pressures met in FPEs promote the survival of *L. monocytogenes* population s and likely impact the genome evolution through HGT of MGEs, acquisition or loss of genes, and to a lesser extent by rare DNA recombination events or point mutations. Plasmid and prophage profiling might be relevant for implementing genomics in advanced predictive modelling of growth and survival of *L. monocytogenes* CCs in RTE food and processing plant environments.

## Methods

### Bacterial strains and seafood facilities characteristics

Keeping in mind the aim of this study, a selection of persistent *L. monocytogenes* subtypes was set up from a strain collection of the department of fishery products and aquaculture (Boulogne-sur-Mer), one site of the Laboratory for Food Safety in the French Agency for Food, Environmental and Occupational Health & Safety (Anses). *L. monocytogenes* contaminated samples were repeatedly collected over 2–6 years from three geographically closed seafood facilities producing RTE foods (Additional file 1): two smoked-fish companies manufacturing smoked-salmon and smoked-herring (plants A and B), and a shrimp-processing company (plant C). Raw salmon and herring were originated from northern European aquaculture industry and captured in the Atlantic sea, respectively, while shrimps were imported from different countries (e.G. *Madagascar* and Vietnam). In the three seafood processing companies, the various working surfaces were mainly constituted of stainless steel, PVC or polyurethane, and the cleaning and disinfection operations (C&D) were performed by service providers with QACs and/or peroxide-based solutions. The isolation of bacterial strains from contaminated samples was originally performed according to standard method EN ISO 11290-1 and − 2 [83] and pulsed-field gel electrophoresis (PFGE) typing with *Apa*I and *Asc*I restriction enzymes was carried out as previously described by Midelet et al. (2007) (Additional file 2). Recurrent pulsotypes from different food products (i.e. shrimp, smoked-salmon and smoked-herring) in the processing line and related working environment (in direct contact and without any contact with foods) were found to be either common to multiple plants or specifically associated to individual plant. Therefore, a panel of 96 strains was selected for WGS according to i) the repeated isolation of indistinguishable pulsotypes in the same facilities over several years, ii) the PFGE profile common to plants A and B and iii) PFGE profiles specific to each plant.

### Genomic DNA extraction and whole genome sequencing

Genomic DNA of selected *L. monocytogenes* strains was extracted using the Wizard® Genomic DNA Purification Kit (Promega, France) according to manufacturer’s instructions for Gram-positive bacteria. Nanodrop® Spectrophotometer and Qbit® fluorimeter were used to assess the quantity of the extracted gDNA. Global integrity of gDNA (200 ng) was assessed via horizontal agarose electrophoresis (Seakem GTG Agarose gel at 0.8% in TBE 1X gel) and the library of genomes were sequenced by paired-end reads (i.e. 2 × 150 bp) at the ‘Institut du Cerveau et de la Moëlle’ (ICM, France, https://icm-institute.org/fr/) using Nextera XT DNA Library Prep Kit and Nextseq500 sequencing system (Illumina).

### De novo assembly and genome annotation

An in-house developed pipeline called ARTwork (Assembly of Reads and Typing workflow) [47] was used for the generation of draft genome assemblies. Briefly, short paired-end reads are decompressed, the coverage of reads is estimated with BBmap [84] to exclude reads lower than 20X, and the others are normalized at 100X with Bbnorm (part of the BBMap v35.85 package; http://sourceforge.net/projects/bbmap). Reads are then quality checked and a report is produced using FastQC v0.11.8 [85] before trimming of adapters and low-quality reads (i.e. minimal length of 50 bp and quality score of 20) with Trimmomatic v0.38 [86] Contigs are therefore generated using the SPAdes v3.9 assembler [87] and the MinHash v2.0 estimation of Jaccard similarity index [88] is applied to identify the closely related reference genome from an internal database of high-quality complete reference genomes. Reference-based scaffolding and gap closure steps are then performed with MeDuSa [89] and GMcloser [90], respectively. Then, scaffolds shorter than 200 bp are trimmed with Biopython and annotation is performed with Prokka v1.13.4 [53]. Finally, a qualitative evaluation of the assembly is then performed by computing various metrics with QUAST [91]. High-quality draft genomes of 94 *L.monocytogenes* strains were obtained with ARTwork pipeline (Additional file 3 and Additional file 4), whose source code is available at https://github.com/afelten-Anses/ARtWORK. FASTQC and QUAST metrics were combined by MultiQC [92].

### MLST-defined clonal complexes (CCs)

The clonal complex (CC) of each draft genome was defined in silico by a 7-gene multilocus sequence typing (MLST) schema (i.e. *abcZ*, *bglA*, *cat*, *dapE*, *dat*, *ldh* and *lhkA*) [93, 94] using the open-source command line MLST software *mlst* v2.16.1.

### Core genome SNPs-based phylogenomic reconstruction

A reference-based variant calling analysis was performed using the recently developed iVARCall2 pipeline [51]. Short reads were mapped against the complete reference genome of *L. monocytogenes* EGD-e (acc. No. NC003210) covering on average 2,789,940 bp (~ 95%). Briefly, after trimming of pair-end reads with Trimmomatic v0.38 (i.e. length > 50 bases and GC > 30), reads are mapped with the Burrows-Wheeler Alignment tool (BWA) v0.7.17 [95] and sorted with Samtools v1.9. Any potential read duplicates are then marked and removed, and a realignment around InDels is performed with the Genome Analysis Toolkit (GATK) v3.7. In particular, the GATK HaplotypeCaller algorithm (which provides local assembly around variant regions) is used for SNPs/InDels calling and filtering in accordance with the best practice [96] for the retaining of high-confidence variants. Finally, SNPs and InDels are stored by the iVARcCall2 workflow in genome Variant Call Format (gVCF) files for each sample, a single VCF file for the whole genome dataset, a single FASTA file of concatenated core variants and another single FASTA file of pseudogenomes. The pseudogenomes were used for the generation of a phylogenomic tree with the IQ-tree software v1.6.7 [97] using the MoledFinder fast model-selection option [98] with an ultrafast bootstrap (UFboot) approximation approach [99] of 1000 bootstrap replicates. The inter- and intra-cluster pairwise SNPs differences were calculated and plotted using the R packages “ggplot2”, “dplyr” and “harrietr”, with a script adapted from the R package harrietr (https://github.com/andersgs/harrietr). In order to support the robustness of the phylogenomic reconstruction, another core genome SNPs-based phylogenomic reconstruction was also performed using Snippy v4.0.2 (https://github.com/tseemann/snippy) implementing Freebayes v1.2.0 [100] as variants caller. The topology and clustering of both trees were compared with the R packages dendextend and phangorn (Additional file 5).

### Pangenomic profile

The pangenome of the 94 *L. monocytogenes* strains was defined using Roary v3.11.2 [52] with a 95% BLASTP identity cut-off. Briefly, the Prokka-annotated genomes in general feature format 3 (GFF3) files were used as Roary input to extract the coding sequences. The latters are filtered (i.e. sequences with more than 5% of Ns or less than 120 bp and/or without start/stop codons are removed) and clustered at different levels to finally compute a matrix of presence and absence of each gene in each isolate. Genes conserved in the 99% of the isolates will represent the “core genes” while the non-core genes (i.e. present in < 99% of genomes) will be considered as “accessory genes”. The pangenome and a neighbourhood joining tree inferred based on the binary matrix of presence and absence of accessory genes produced by Roary were visualized on Phandango v1.3.0 (http://jameshadfield.github.io/phandango/#/) along with associated metadata. This tool allowed to interactively screen the presence of genetic markers relevant for unravelling the persistence of *L. monocytogenes* in FPEs (e.g. plasmid- and transposons-associated resistance determinants) and their variations between strains.

### Enrichment of genes from each plant

Investigating the differences in genes composition can lead to a better understanding of key processes such as selection and adaptation. Therefore, a gene enrichment analysis was performed using Scoary v1.6.16 [64] to identify patterns of genes significantly overrepresented in isolates from each plant (A, B and C). The Roary matrix and a pre-defined trait representing the studied plant of each isolate were used as Scoary input. The *collapse* flag was used in the enrichment analysis to avoid multiple comparison corrections of identically distributed genes in the sample, such as plasmid and prophage genes, which would not add any information individually. Patterns of loci were considered strongly associated to plant A, B and C if the Benjamini-Hochberg (BH)-corrected *p*-value was less than 2 × 10^− 5^ and the specificity > 99% (i.e. almost exclusively present in trait-positive isolates). Manual refinement of gene annotations and assessment of the synteny of associated loci were carried out through Artemis software [101] and searching homology with BLASTn tool from NCBI [102].

### Plasmidome reconstruction

The plasmidome (i.e. total plasmid populations in a given environment) of the 94 *L. monocytogenes* strains was de novo predicted using a combination of the bioinformatics tools plasmidSPAdes [58] and MOB-suite [59]. As first, the plasmidSPAdes algorithm was used with default settings for extracting and assembling of putative plasmids from short reads, mainly based on differential estimations of median coverage from chromosomal and plasmid sequences. Then, the recently developed MOB-suite software was used to predict the transmissibility of the plasmidSPAdes assembled sequences. Based on the presence of relaxase, mate-pair formation and *oriT* sequences, the plasmid is classified as “conjugative” (i.e. if it includes both a relaxase and a mate-pair formation marker), “mobilizable” (i.e. if it includes only a relaxase or an *oriT*) and “non-mobilizable” (i.e. if both a relaxase and an *oriT* are missing). Moreover, the MOB-recon algorithm was used to identify plasmidic contigs from the draft genomic assemblies generated by ARTwork in order to confirm the performance of plasmidSPAdes assembler. The BLAST-based MOB-recon tool uses markers from sequence databases of known replicons and relaxase in conjunction with a reference database of clustered plasmids provided by MOB-cluster [59]. Finally, the PLSDB web-resource [60], a comprehensive large-scale database including 13,789 (November 2018) bacterial plasmid complete sequences, was used for a large-scale comparative analysis to retrieves any plasmid records (and its meta-information) similar to the herein assembled plasmids. The PLSDB database was interrogated using Mash [88] (i.e. the command *dist*) with a maximal *p*-value and distance thresholds set to 1 × 10^− 1^. Plasmids genome map and comparison were visualized using EasyFig v2.2.3 [103] and BRIG v1.0 [104].

### Prediction of prophages

In order to identify the putative prophage sequences harboured by the *L. monocytogenes* strains analysed in this study, the 94 draft genomes were submitted through the URL API to the PHASTER (PHAge Search Tool –Enhanced Release) server [63]. This application scores prophage regions as “intact”, “questionable” or “incomplete” based on several criteria such as the number of CDSs homologous to certain phages, the percentages of CDSs that match a certain phage (i.e. major potential phage), the size of the phage region and specific phage-related keywords (e.g. tail, head, capsid, portal, terminase, integrase, etc). The functional annotations of CDSs homologous to known phages are also provided. Intact and questionable regions with sequence length over 20 kbp were considered for the prophage profiling of the genome dataset and the average nucleotide identity (ANI) computed for each genome pairwise using FastANI v2.0 (https://github.com/ParBLiSS/FastANI).

## Supplementary information

**Additional file 1. **List of 94 *L. monocytogenes* isolates included in the present study, associated metadata and sequence accessions.

**Additional file 2.** Characterization of selected strains through pulsed-field gel electrophoresis.

**Additional file 3.** Overview of the genomes, CC, and quality parameters of de novo assembly and read mapping.

**Additional file 4 **Quality metrics of mapping (i.e. iVARcall2) and de novo assembly (i.e. ARTwork) from the studied *L. monocytogenes* genomes.

**Additional file 5.** Topology and distance comparison between phylogenomic reconstructions based on variant calling analyses from iVARCall2 and Snippy pipelines.

**Additional file 6 **Accessory genes-based Neighbour-joining clustering and pangenome matrix of 94 *L. monocytogenes* isolates.

**Additional file 7.** List of plasmids, prophages and transposons.

**Additional file 8.** Gene content of pLM80-like and pLM6179-like plasmids.

**Additional file 9.** List of prophages per genome and insertion sites, size, number of CDS and annotation.

**Additional file 10.** Pairwise comparison matrix of prophage sequences.

**Additional file 11.** List of genes enriched in the originating plant.

## Data Availability

All the short pair-end reads of the 94 *Listeria monocytogenes* included in this study have been submitted to the European Nucleotide Archive (http://www.ebi.ac.uk/ena) and are available under the study accession No: PRJEB30603.

## Abbreviations

As: Arsenic
BH: Benjamini-Hochberg
CC: Clonal Complexes
Cd: Cadmium
C&D: Cleaning and disinfection
CDS: Coding DNA sequence
EU: European Union
FPE: Food processing environment
GFF: general feature format
gVCF: genome variant call format
HGT: Horizontal gene transfer
LGI: Listeria genomic island
MGE: Mobile genetic element
ML: Maximum-Likelihood
MLST: multilocus sequence typing
NCBI: National Center for Biotechnology Information
NJ: Neighbour-joining
RTE: Ready-to-eat
SNP: Single nucleotide polymorphism
ST: Sequence type
tRNA: Transfer RNA
WGS: Whole Genome Sequencing
