# Supplementary material for: Dynamics of mobile genetic elements of Listeria monocytogenes persisting in ready-to-eat seafood processing plants in France
Source: BMC Genomics. 2020 Feb 6;21:130. doi: 10.1186/s12864-020-6544-x (PMC7006209; doi:10.1186/s12864-020-6544-x)
Supplement: Supplementary file 2 — Additional file 2. Characterization of selected strains through pulsed-field gel electrophoresis. [file 12864_2020_6544_MOESM2_ESM.pdf]

Additional file 2: Characterization of selected bacterial strains through pulsed-field gel electrophoresis

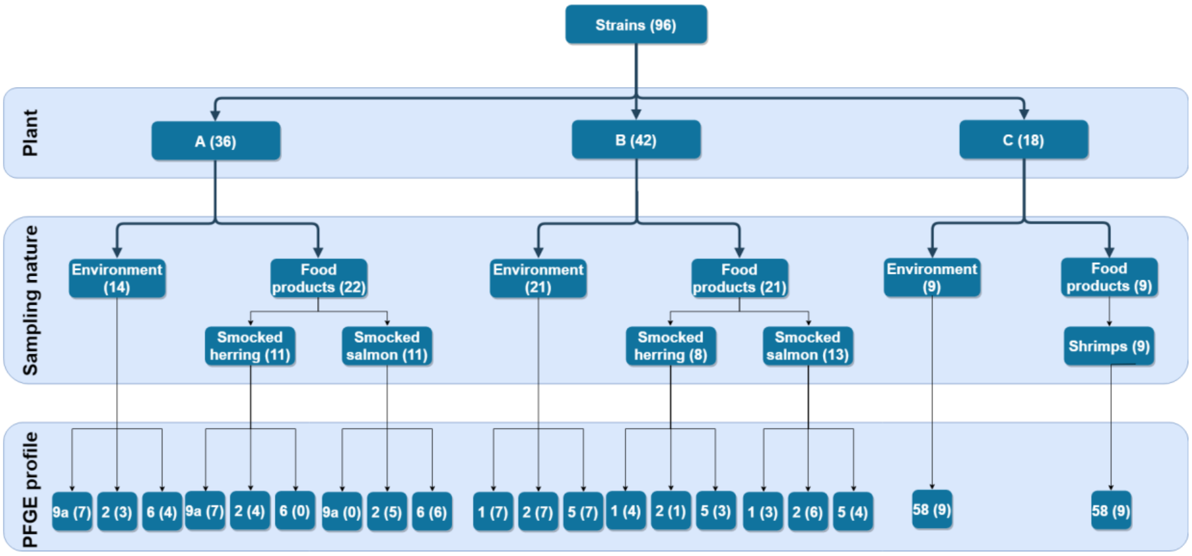

Pulsed-field gel electrophoresis (PFGE) typing was performed on all the *Listeria monocytogenes* strains from the collection of the Laboratory of seafood products (Boulogne-sur-Mer, France). Both *Apa*I and *Asc*I enzymes for restriction digestion were used as previously described by Midelet et al. (2007) for the pulsotype determination [1]. Electrophoretic patterns were analysed using Bionumerics software v 6.5 (Applied Maths, Sint-Martens-Latem, Belgium). The DNA bands generated by single and combinations of restriction enzymes were analysed using Dice's coefficient with a maximum position tolerance of 1.2% and the PFGE profile assigned to each strain. Some PFGE profiles (e.g. profile 1, 5, 6, 9a and 58) were found to be specific to each company while other (e.g. profile 2) common to plants A and B. Moreover, the same PFGE profile was repeatedly isolated over time from different food matrices (i.e. salmon and herring) as well as from environmental sites even after C&D. The information about the genotype and its recurrence in the processing plants and in the samples of different nature was considered for the selection of 96 *Listeria monocytogenes* strains for whole genome sequencing.

References

1. Midelet-Bourdin G, Leleu G, Malle P. Evaluation of the international reference methods NF EN ISO 11290-1 and 11290-2 and an in-house method for the isolation of Listeria monocytogenes from retail seafood products in france. J Food Prot. 2007;70:891–900.
