# Supplementary material for: Dynamics of mobile genetic elements of Listeria monocytogenes persisting in ready-to-eat seafood processing plants in France
Source: BMC Genomics. 2020 Feb 6;21:130. doi: 10.1186/s12864-020-6544-x (PMC7006209; doi:10.1186/s12864-020-6544-x)
Supplement: Supplementary file 4 — Additional file 4 Quality metrics of mapping (i.e. iVARcall2) and de novo assembly (i.e. ARTwork) from the studied L. monocytogenes genomes. [file 12864_2020_6544_MOESM4_ESM.pdf]

Additional file 4. Quality metrics of mapping (i.e. iVARcall2) and *de novo* assembly (i.e. ARTwork) from the studied *Listeria monocytogenes* genomes

An essential step in comparative genomics based studies is the Quality Control (QC) of WGS data in order to guarantee the accuracy of sequencing results obtained by *in silico* genome-wide analysis. Poor quality of read sequences as well as contamination of DNA can lead to significant errors in variant calling (low-depth of sequencing impact on false-positive rate) and gene prediction analyses [1]. Even though the harmonization and standardization of WGS data analysis is still an ongoing process [2], a number of metrics for QC of *de novo* draft genome (i.e. contiguity of assemblies) and genome coverage (i.e. number of reads mapped to a specific position within the reference genome, so called "mappability") is currently available [3–5]. In this study, standard quality metrics of reads mapping (i.e. iVARcall2) and *de novo* assembly (i.e. ARTwork) obtained from Illumina paired end reads of 96 *Listeria monocytogenes* genomic DNA were assessed and reported in the boxplots. In particular, the quality of reads mapping onto the reference genome was evaluated based on the depth of coverage (average number of times that a base of a genome is sequenced) and breadth of coverage (percentage of bases of a reference genome that are covered with a certain depth). Moreover, contiguity measures, such as the size of assembled genomes (express in total number of bases and representing an indicator of exogenous DNA contamination), and the total number of contigs/scaffolds along with the N50 value (size of the largest contig, or scaffold, for which half the total size is contained in that contigs and those larger), were calculated. Overall, high values of depth and breadth of coverage (1st and 3rd quartile = 145-426X and 94-96%, respectively) have been estimated confirming the high quality of the Illumina short reads for further analyses. Accordingly, genome sizes as well as the number of scaffolds and the N50 (median values of 3,021,803 bp, 4 and 2,969,304 bp, respectively) demonstrated high performance of ARTwork pipeline in term of high contiguity of *de novo* assemblies for almost all *L. monocytogenes* samples (~98%). However, two out of 96 assemblies were discarded from further analyses since suspected of contamination (total genome length higher than 3.8 Mbp and number of

scaffolds > 354).

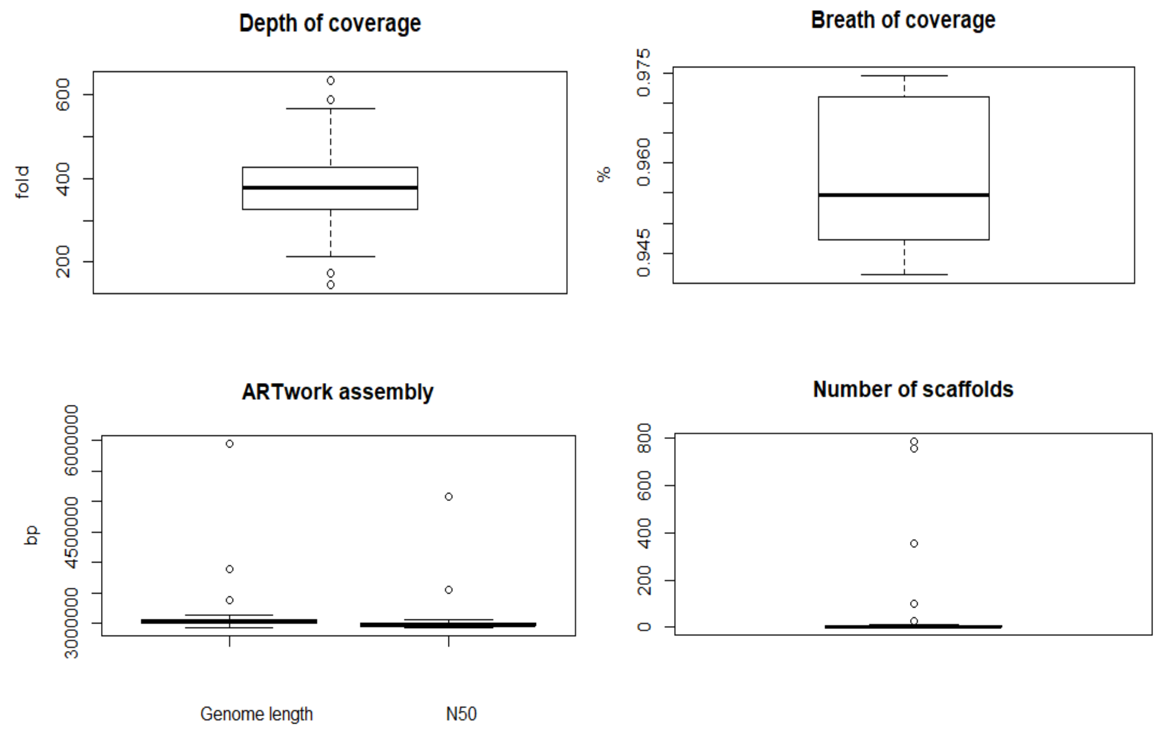

References

1. Sims D, Sudbery I, Ilott NE, Heger A, Ponting CP. Sequencing depth and coverage: key considerations in genomic analyses. Nature Reviews Genetics. 2014;15:121–32.

2. Revez J, Espinosa L, Albiger B, Leitmeyer KC, Struelens MJ, ECDC National Microbiology Focal Points and Experts Group. Survey on the Use of Whole-Genome Sequencing for Infectious Diseases Surveillance: Rapid Expansion of European National Capacities, 2015-2016. Front Public Health. 2017;5:347.

3. Ellington MJ, Ekelund O, Aarestrup FM, Canton R, Doumith M, Giske C, et al. The role of whole genome sequencing in antimicrobial susceptibility testing of bacteria: report from the EUCAST Subcommittee. Clinical Microbiology and Infection. 2017;23:2–22.

4. Gurevich A, Saveliev V, Vyahhi N, Tesler G. QUAST: quality assessment tool for genome assemblies. Bioinformatics. 2013;29:1072–5.

5. Lüth S, Kleta S, Al Dahouk S. Whole genome sequencing as a typing tool for foodborne pathogens like Listeria monocytogenes – The way towards global harmonisation and data exchange. Trends in Food Science & Technology. 2018;73:67–75.
