# Supplementary material for: Dynamics of mobile genetic elements of Listeria monocytogenes persisting in ready-to-eat seafood processing plants in France
Source: BMC Genomics. 2020 Feb 6;21:130. doi: 10.1186/s12864-020-6544-x (PMC7006209; doi:10.1186/s12864-020-6544-x)
Supplement: Supplementary file 5 — Additional file 5. Topology and distance comparison between phylogenomic reconstructions based on variant calling analyses from iVARCall2 and Snippy pipelines. [file 12864_2020_6544_MOESM5_ESM.pdf]

**Additional file 5. Topology and distance comparison between phylogenomic reconstructions based on iVARCall2 and Snippy pipelines**

Concatenated core variants from the reference alignments of short reads of 94 *L. monocytogenes* isolates were generated using iVARCall2 and Snippy pipelines, including respectively GATK-HaplotypeCaller and Freebayes, two of the most widely used variant callers for the identification of SNPs and InDels in microbial genomics study. Then, the phylogenomic trees built with the iVARCall2- and Snippy-based concatenated SNPs (50,349 and 66,142 SNPs respectively) were compared in terms of tree topologies (i.e. patterns of branching) and distances (i.e. branch lengths). The topology variations and indexes for distance were calculated using different R packages in accordance to Henri et al. (2017) [1]. In particular, the Fowlkes-Mallows index (FMI) [2] for the measure of similarity between two clusterings was computed. Moreover, the correlation between the cophenetic distance matrices of the two cluster trees was computed using the cophenetic correlation coefficient (CCC) [3] implemented in the "cor_cophenetic" function of R "dendextend" package. To better visualize the comparison between the two phylogenies reconstructions, data were plotted connecting the same strains between leaves (on the left iVARCall2- based and on the right Snippy-based trees). The comparison of trees topology, calculated calibrating the trees by correlating sampling date to the tip distance from root, revealed that the two variant calling pipelines lead to highly similar results showing no clustering differences in the shapes of the trees. Based on the FMI and CCC estimation, the quantification of similarity between the iVARCall2- and Snippy-based phylogenies resulted in extremely high values, FMI = 1 and CCC = 0.9985065. These statistical results strongly support the conclusion that these two variant calling methods lead to the same phylogenomic reconstruction, even though the number of analysed variant sites differed. Therefore, results from iVARCall2 pipeline were used for further analyses in the present study.

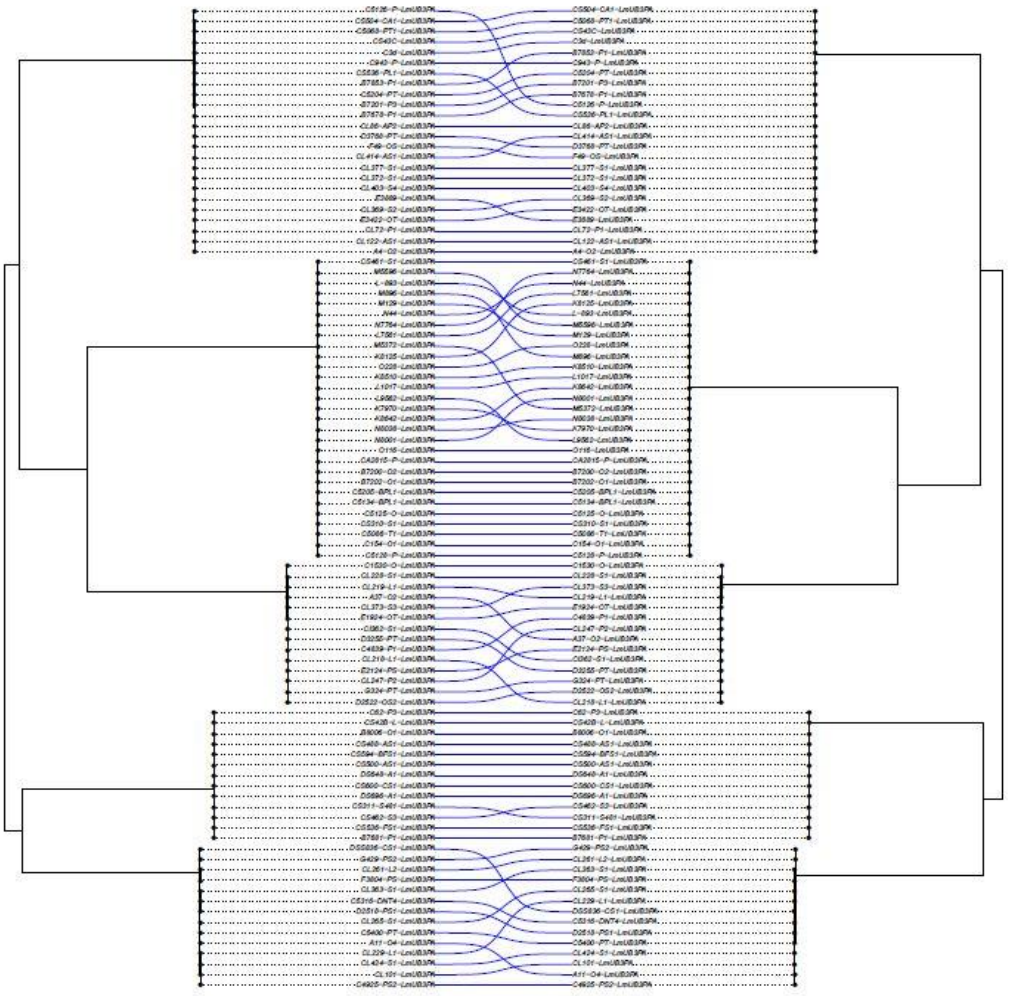

## References


1. Henri C, Leekitcharoenphon P, Carleton HA, Radomski N, Kaas RS, Mariet J-F, et al. An Assessment of Different Genomic Approaches for Inferring Phylogeny of Listeria monocytogenes. Front Microbiol. 2017;8.

2. Fowlkes EB, Mallows CL. A Method for Comparing Two Hierarchical Clusterings. Journal of the American Statistical Association. 1983;78:553–69.

3. Sokal RR, Rohlf FJ. The Comparison of Dendrograms by Objective Methods. Taxon. 1962;11:33–40.
