# Supplementary material for: Dynamics of mobile genetic elements of Listeria monocytogenes persisting in ready-to-eat seafood processing plants in France
Source: BMC Genomics. 2020 Feb 6;21:130. doi: 10.1186/s12864-020-6544-x (PMC7006209; doi:10.1186/s12864-020-6544-x)

Additional file 6. Accessory genes-based Neighbour-joining clustering and pangenome matrix of 94 *L. monocytogenes* isolates

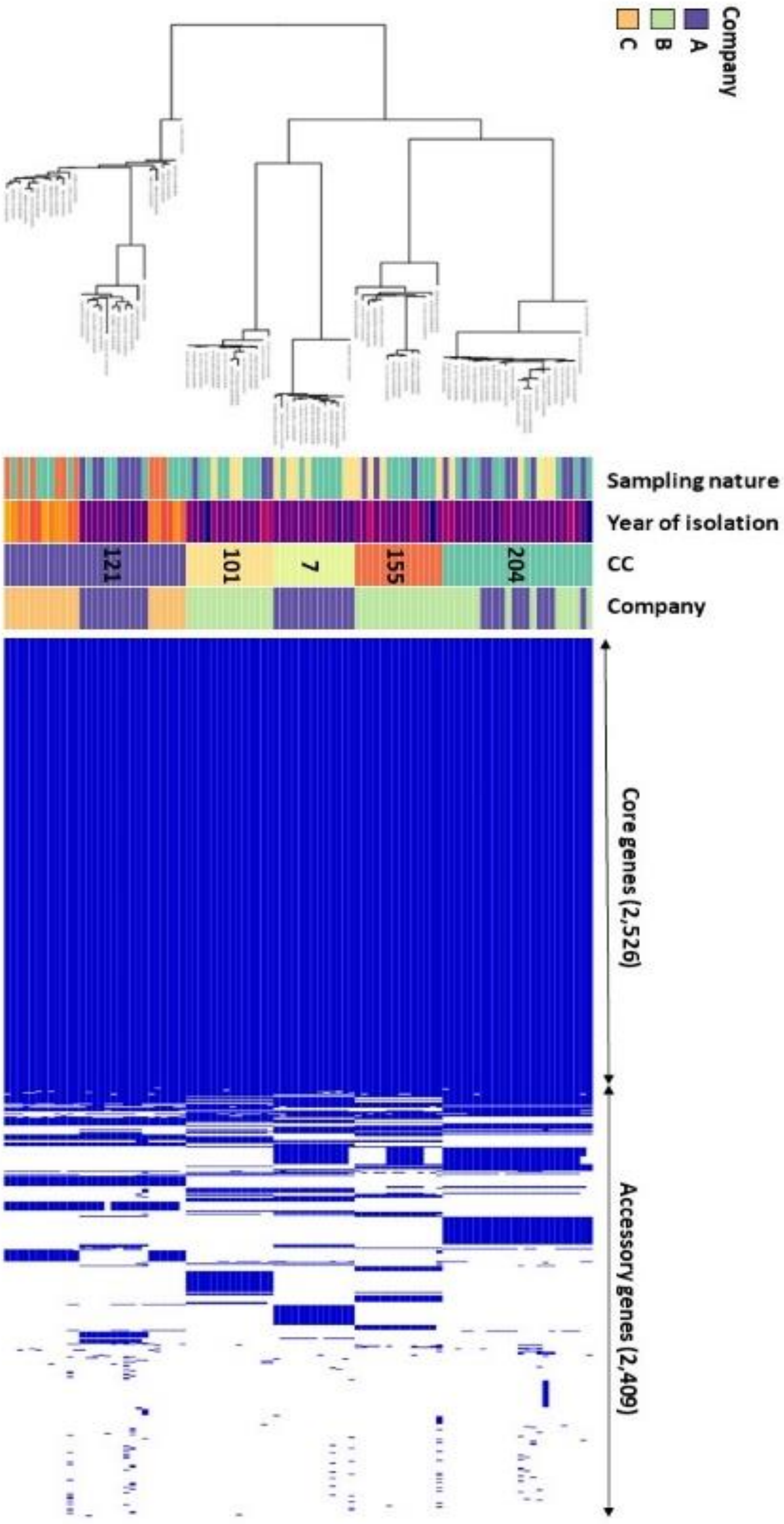

Supplement: Supplementary file 6 — Additional file 6 Accessory genes-based Neighbour-joining clustering and pangenome matrix of 94 L. monocytogenes isolates. [file 12864_2020_6544_MOESM6_ESM.pdf]
