# Supplementary material for: Dynamics of mobile genetic elements of Listeria monocytogenes persisting in ready-to-eat seafood processing plants in France
Source: BMC Genomics. 2020 Feb 6;21:130. doi: 10.1186/s12864-020-6544-x (PMC7006209; doi:10.1186/s12864-020-6544-x)
Supplement: Supplementary file 8 — Additional file 8. Gene content of pLM80-like and pLM6179-like plasmids. [file 12864_2020_6544_MOESM8_ESM.pdf]

Additional file 8. Main genetic component of pLM80-like and pLM6179-like plasmids

The gene encoding for VirB protein from type IV secretion system along with traG characterized the conjugative system of plasmids from CC121 whereas traCDG and genes encoding for putative conjugal transfer protein described the mobility of pLM80-like plasmids. Genes encoding for Tra-family proteins involved in plasmid conjugal transfer such as traG, have been already described in *L. monocytogenes* [1]. Moreover, all plasmids detected in this study contained a number of transposable elements (TEs) such as Tn3, Tn7, Tn5422 and Tn522 family transposases, along with several insertion sequences (ISs) such as IS3, IS6, IS1216 and IS30 flanking the stress-tolerance genes. In addition to disinfectants and heavy metals resistance genes described above, genetic features like the copper-responsive transcriptional repressor CopY [2], the DEAD/DEAH-box helicase conferring resistance to low temperature growth [3], the oxidative-stress-inducible qorB encoding quinone oxidoreductase and the RavA ATPase for response to acid stress conditions, were included in the pLM80-like plasmids. Several genes predicted in pLM80-like plasmids encoded mechanisms playing an important role in protecting cells against oxidative stress. In particular, a glycine betaine/carnitine transport binding protein, GbuC, for hyperosmotic stress reduction [4]; a Zinc-transporting ATPase, zosA, for the hydrolysis of ATP with the transport of zinc into the cell; a gene encoding putative NADH peroxidase (npr) shown to reduce hydrogen peroxide in Lactobacillus casei [5]; and the Chaperone protein ClpB involved in general stress tolerance but also in virulence of *L. monocytogenes* in food samples [6]. More recently, Naditz et al. (2019) analysed the differences between wild type and plasmid-cured *L. monocytogenes* strains exposed to stress conditions and described the plasmid-encoded ClpB protein as possibly contributing to increase survival to heat stress at 55 ˚C. A different protease of the Clp-family (ClpC subunit) sharing the same domain that contains an ATP-binding site is encoded in the plasmids from CC121. Proteins from this superfamily are thought to protect cells from stress by controlling the aggregation and denaturation of vital cellular structures and recently shown to significantly increase resistance to heat shock in *L. monocytogenes* [7]. The plasmids from CC121 were also characterized by the presence of a Toxin-Antitoxin (TA) systems composed of closely linked genes (e.g. mazF/chpS) encoding a toxic and

an antitoxin protein. The plasmid-encoded TA systems appears to be involved in the plasmid maintenance in daughter cells as well as in plasmid-plasmid competition through a post-segregational killing or addiction mechanism, relying on the inhibition of the target cell growth and/or survival due to the toxin-target interaction, once the toxin is released from the TA complex [8].

References

1. Bakker HC den, Bowen BM, Rodriguez-Rivera LD, Wiedmann M. FSL J1-208, a Virulent Uncommon Phylogenetic Lineage IV Listeria monocytogenes Strain with a Small Chromosome Size and a Putative Virulence Plasmid Carrying Internalin-Like Genes. Appl Environ Microbiol. 2012;78:1876–89.

2. Rademacher C, Masepohl B. Copper-responsive gene regulation in bacteria. Microbiology. 2012;158:2451–64.

3. Chan YC, Wiedmann M. Physiology and Genetics of Listeria Monocytogenes Survival and Growth at Cold Temperatures. Critical Reviews in Food Science and Nutrition. 2008;49:237–53.

4. Angelidis AS, Smith GM. Three transporters mediate uptake of glycine betaine and carnitine by Listeria monocytogenes in response to hyperosmotic stress. Appl Environ Microbiol. 2003;69:1013–22.

5. Serata M, Iino T, Yasuda E, Sako T. Roles of thioredoxin and thioredoxin reductase in the resistance to oxidative stress in Lactobacillus casei. Microbiology. 2012;158:953–62.

6. Mujahid S, Pechan T, Wang C. Protein expression by Listeria monocytogenes grown on a RTE-meat matrix. Int J Food Microbiol. 2008;128:203–11.

7. Pöntinen A, Aalto-Araneda M, Lindström M, Korkeala H. Heat Resistance Mediated by pLM58 Plasmid-Borne ClpL in Listeria monocytogenes. mSphere. 2017;2. doi:10.1128/mSphere.00364-17.

8. Guglielmini J, Van Melderen L. Bacterial toxin-antitoxin systems. Mob Genet Elements. 2011;1:283–90.
